# Simulated co-optimization of renewable energy and desalination systems in Neom, Saudi Arabia

**DOI:** 10.1038/s41467-022-31233-3

**Published:** 2022-06-18

**Authors:** Jefferson A. Riera, Ricardo M. Lima, Ibrahim Hoteit, Omar Knio

**Affiliations:** 1 Physical Science and Engineering Division, King Abdullah University of Science and Technology, (KAUST), Thuwal, 23955-6900 Saudi Arabia; 2 Computer, Electrical and Mathematical Sciences & Engineering Division, King Abdullah University of Science and Technology, (KAUST), Thuwal, 23955-6900 Saudi Arabia

**Keywords:** Energy modelling, Environmental sciences

## Abstract

The interdependence between the water and power sectors is a growing concern as the need for desalination increases globally. Therefore, co-optimizing interdependent systems is necessary to understand the impact of one sector on another. We propose a framework to identify the optimal investment mix for a co-optimized water-power system and apply it to Neom, Saudi Arabia. Our results show that investment strategies that consider the co-optimization of both systems result in total cost savings for the power sector compared to independent approaches. Analysis results suggest that systems with higher shares of non-dispatchable renewables experience the most significant cost reductions.

## Abstract (web summary)

Co-optimization of renewable power and water desalination systems for Neom, a futuristic seaside city in an arid region, results in more pronounced cost investment savings for a high share of renewable sources.

## Introduction

The water and energy sectors are becoming inextricably linked as water resources become scarcer worldwide^1^. In 2014, nearly 4% of global electricity consumption was due to the water sector. Projections estimate that the energy used by the water sector will double by 2040^2^. The largest increase is associated with desalination, an energy-intensive process that requires electrical and/or thermal power to produce freshwater. Today, desalination and water re-use meet only 0.7% of the global water need; however, these processes account for nearly a quarter of the total energy consumption by the water sector^2^.

Desalination technology has been commonly used in the Middle East and North Africa (MENA), where surface freshwater resources are limited, and fossil groundwater is rapidly being depleted. Countries in the MENA region have increased their desalination capacity to keep up with water demand and reduce groundwater withdrawals^3^. In 2016, approximately 7 billion m^3^ of water were desalinated in the MENA region, a number that is expected to increase twelve-fold to eliminate reliance on non-renewable groundwater extraction^2^. Saudi Arabia, which experiences the largest water deficit in the region, will see the largest growth in desalination and water storage capacity. The country is expected to desalinate nearly 30 billion m^3^/day of water in 2040, up from 1.5 billion m^3^/day in 2014^4^.

While desalination plays a significant role in the Middle East region, it has gained prominence in other parts of the world, including the United States, where droughts have dwindled water supplies^5^. Water-rich states located in the central region of the US have, for decades, provided water across borders to meet the growing demand of the dry Southwest. To do so requires transportation networks with significant investment and high operational costs. Similarly, geopolitics and concerns over aquifer recharge in the central states may limit water sent to dry areas^6^.

With the risk of permanent aridification of the Southwest states, the region has begun to invest heavily in desalination technology rather than relying on neighboring states to meet water demand. In 2015, the Carlsbad Desalination Plant, the largest desalination plant in the United States with a capacity of 190,000 m^3^/day, opened in California^7^. Other states such as Arizona and Texas have followed suit to address their water scarcity issues. Currently, the United States has a total installed desalination capacity of 7.5 million m^3^/day^6^.

The impact of growing desalination demand on the power sector is significant. Electricity consumption due to desalination is expected to grow ten times the current consumption in MENA by 2050^3^. Similar trends are found in other parts of the world^1^. These changes are not only associated with growth in desalination needs but also due to a shift away from thermal-based desalination^2^. The alternative approach, electric-based desalination, relies on electric pumps to push seawater through membranes to remove the salt. Such an approach is deemed a sustainable method to desalinate water if the electricity is derived from renewable sources.

New projects in the region are pushing the integration of desalination with renewable technologies to ensure a sustainable stream of water. Such projects include the futuristic cities/communities of Masdar City in the United Arab Emirates and Neom^8^ in Saudi Arabia. The idea of futuristic cities is to create environments dedicated to sustainable practices such as zero waste, high renewable energy penetration (zero carbon) and carbon-neutral fuels^9^, and green infrastructure. With these considerations in mind, it is essential to develop modeling and optimization tools to understand the impact of a growing water sector on the power system.

Tools such as generation expansion planning (GEP) models enable decision-makers to identify the optimal generation/production mix of a system^10^. Generation expansion planning models have evolved^11^ and become more important as power systems transition to integrate renewable generation facilities^10,12–19^, energy storage^16^, and required transmission expansion^17,19^, accommodate environmental costs^10^, and address reliability and flexibility concerns. The integration of investment and operational constraints in GEPs provides a means to determine the best course of action regarding what technologies to install, the plant capacities, or where and when to build them^20^. These models are driven by technology costs and renewable resource availability to meet power demand.

GEPs are challenging to solve because they involve decisions happening at different time scales. Operational decisions typically occur hourly for the power sector, whereas investment decisions occur yearly or after several years^11^. Such models become harder to solve when dealing with variabilities such as renewable power output, intra-annual demand variability, or long-term uncertainty such as annual demand growth, investment costs, or operating cost^17^. It is necessary to consider that when dealing with uncertainty, longer time horizons result in higher uncertainty.

GEPs have been historically used to model an individual sector, particularly the power sector^10,12–19^. As sectors become more interdependent, there has been growing research interest in sector coupling and co-optimization to understand the reliability and flexibility of such systems. With co-optimization, sectors can react to one another to make more informed investment and operational decisions. On the operational side, electricity consumption peaks can be shifted by considering incentives/disincentives on the consumer end (the other sector), a practice known as demand-side management^21^.

In the traditional sense, demand-side management (DSM) refers to strategies and initiatives that encourage consumers to shift their energy use. DSM has two main benefits; first, consumers can benefit from favorable tariffs when their behavior is altered to adjust when and how much electricity they use. Secondly, the power system benefits by shifting energy consumption from peak to non-peak hours, preventing the system from potentially overloading^22^. Demand-side management benefits can also be assessed by shifts in production from high production cost hours to low-cost hours. In investment models, shifting electricity peaks may allow for cost savings by reducing the total power generating capacity necessary to meet demand^23^.

The goal of co-optimizing the water and energy sectors is to exploit potential investment and operational cost savings arising from the optimized sizing of generation plants and storage due to desalination-induced demand-side management. In a traditional power system GEP model, electricity consumption from desalination is accounted for in the total hourly demand at each node, with assumptions made regarding the power consumption by the water sector.

Literature on GEP is vast; however, literature that focuses on the co-optimization of the water and power sectors is limited. Bognar et al.^24^ quantify the cost reductions of water and electricity due to the integration of desalination with a wind-diesel power system on a Cape Verdean island. Caldera et al.^25^ explore the investment strategy of desalination and renewables in Saudi Arabia’s power sector. They found a 1-3% decrease in the annual levelized cost of the integrated system. However, in the previous references, the models are deterministic. Therefore, they do not consider uncertainties in long-term demand growth.

Al-Nory and Brodsky^26^ examine the optimal scheduling of a desalination plant with a smart power grid to provide a buffer for times when renewable resources are interrupted. They found that desalination plants over-produced water when electricity prices were at the marginal level. When electricity prices are high, desalination output decreases, and demand is met primarily with stored water. That study, however, did not explore capacity investments, given that the considered time horizon is only one day. Al-Nory and El-Beltagy^27^ use polynomial chaos expansion to account for uncertainty (renewable supply, water, and electricity demand). However, their planning time horizon is only seven days with a daily resolution. While short-term uncertainty is considered, long-term uncertainties such as demand growth or investment costs are ignored.

This work proposes a multiperiod (2020–2029) generation and expansion planning co-optimization model for a system that considers investment and operation decisions for the power and water sectors under uncertainty. The model accommodates a high penetration of renewable energy sources and introduces flexibility through water storage, batteries, and demand-side management. We assess the benefits of co-optimizing the water and electricity sectors by comparing the results to stand-alone models that optimize the water and power sectors, each acting independently of the other. In a co-optimized model, the electricity demand at each hour excludes desalination consumption – we call this baseline power demand. The water sector has an hourly water demand schedule and must desalinate to meet this demand. The significance of co-optimization is that the two sectors can interact and make cost-saving decisions. Accordingly, the water sector may desalinate water based on electricity production costs or the availability of renewable resources. The power sector must produce enough electricity to meet the baseline demand and the power demand required by the water sector, shifting water production to non-peak electricity hours. The model was applied to Neom, Saudi Arabia, as it is a new construction project – greenfield – which aims to be fully/or near fully renewable. In a particular case, the model includes a 4 GW transmission connection to the existing Saudi Arabian power grid to study the costs and added flexibility to the power-water coupled system. With the same goal in mind, the model also allows for CCGT investments.

## Results

We present a case study to highlight a clear example of the benefits of co-optimization. We choose Neom, Saudi Arabia^8^. The area of Neom is slightly smaller than the country of Belgium; therefore, the implementation of our model takes a country-level perspective, and it can be applied to other countries as well.

Neom is projected (high scenario estimate) to consume nearly 45.35 TWh of electricity by 2030 for a population of 770 thousand people; this will translate to an annual per capita electricity consumption of 59.35 MWh. The projected per capita consumption will be higher than any developed country, including Iceland, at 55.05 MWh. Industries that will contribute to Neom’s high per capita power consumption include renewable tech manufacturing, green chemical production, desalination, and data centers. We follow a scenario-based approach to capture the uncertainty in demand growth each year. The uncertainty is characterized by a set of distinct realizations. Each scenario defines the number of decisions that must be made. Our model considers three scenarios: low, medium, and high, each with a definite demand for water and power and a probability of occurrence. We do not consider uncertainty in renewable resources due to the difficulty of generating hourly forecasts for an entire year or more. However, we used ten years of high-resolution data to generate representative days that represent clusters of days with similar attributes.

**Fig. 1:**
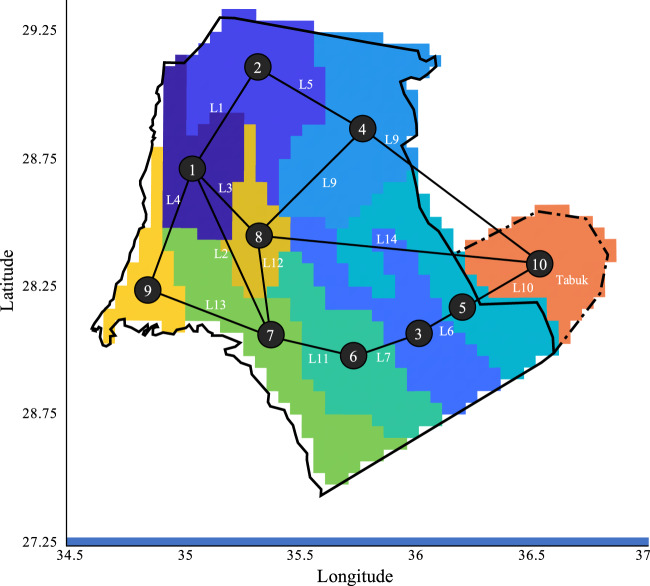
Nodal system for Neom. Generation technologies can be built on ten nodes and linked via 14 possible transmission lines (L1-L14).

We divide Neom into nine nodes; we also include one node located outside of Neom and surrounding the city of Tabuk, as depicted in Fig. 1. Power demand is considered for one node (Node 7), given that most industries and residential areas would be in that location. Having a single demand node could limit the model to building all power generating and water producing capacities at that given node. However, that is only the case if the renewable resource potential for that node is equivalent to or better than all surrounding nodes. Therefore, an expansion would depend on the resource availability of renewables in the regions surrounding the demand node. If desalination facilities are built in other regions along the Red Sea coastline (Nodes 1 and 9), their electricity consumption will create power demand in the nodes where they are constructed. We assess the economics of investing in four possible power generating technologies: combined cycle gas turbines (CCGT), photovoltaics (PV), concentrated solar power (CSP), and onshore wind turbines, as well as battery storage and hydroelectric pumped storage (HPS). We consider constructing 14 possible transmission lines with a nodal balance to ensure that supply meets demand.

For the water sector, reverse osmosis (RO) desalination and water storage tanks are considered for investments. We do not account for water flow at a nodal level but instead define a constraint requiring that the aggregate hourly water production equal the aggregate hourly water demand.

Given that desalination can account for a large percentage of the electricity consumption in the Middle East, we present a power mix where desalination accounts for 20% of the total electricity consumption. We have also included results for a power mix where desalination accounts for 4% of the total electricity consumption in the Supplementary Information under Supplementary Figs. 9–14 and Supplementary Tables 11–17.

We present results for four cases: 1) Base case, where investment is allowed in only renewable generating technologies (CCGT is not considered), 2) the Kingdom of Saudi Arabia (KSA) Grid, where Neom is allowed to obtain power from the existing KSA power network via a 4-GW transmission line, 3) Photovoltaics (PV) Only, where investments are only allowed in PV solar technology and battery storage, and 4) Wind Only, where investments are only allowed in wind turbines and battery storage. The PV Only and Wind Only cases are used to validate our results and understand why wind was selected over PV by our model in the Base case. Furthermore, given Saudi Arabia’s high potential for solar energy, we sought to quantify the decision strategy for systems that are solely powered by PV. In Supplementary Figs. 3 and 4, we present results where we allow for the investment of CCGT under varying levels of renewable penetrations. These scenarios were designed to explore the added flexibility of allowing investments in fossil-fuel-based power generators.

For each case, we compare two strategies: (1) an independent approach, where the power and water sector investments are planned individually, and (2) a co-optimized approach where both sectors are aware of their corresponding decisions and adjust accordingly.

**Fig. 2:**
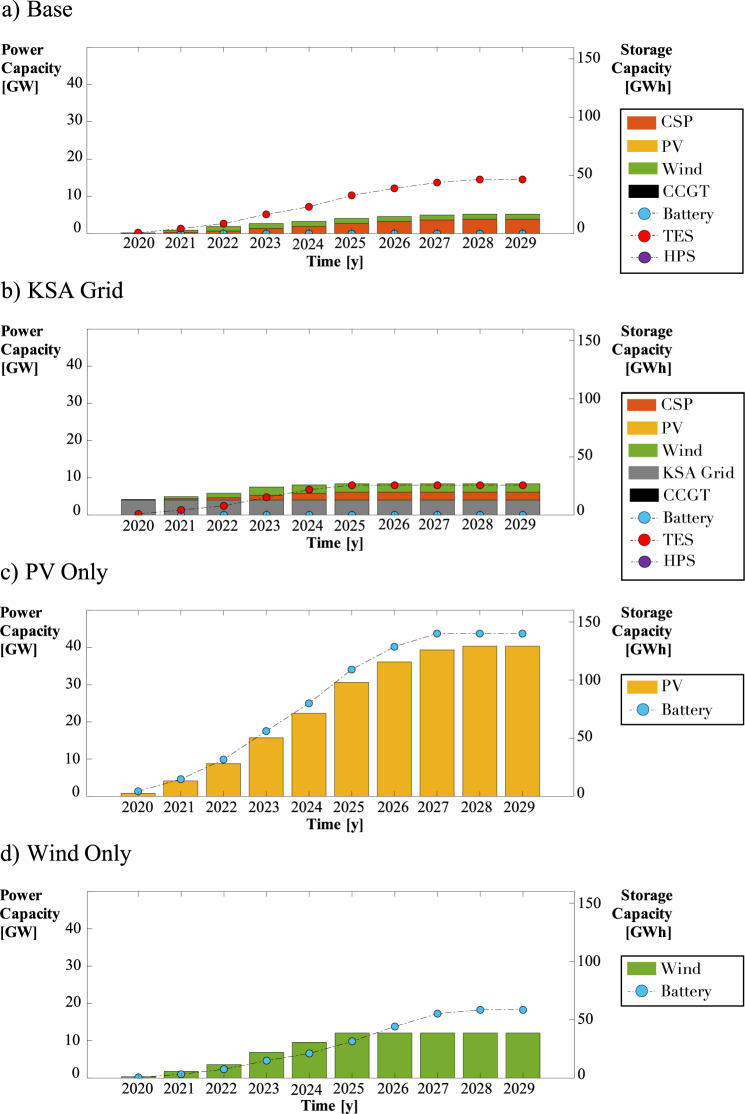
Power generating capacities for the **a**) Base, **b**) the Kingdom of Saudi Arabia (KSA) Grid, **c**) Photovoltaics (PV) Only, and **d**) Wind Only cases. Cases PV Only and Wind Only, without dispatchable generators, require significantly larger generating capacities and battery storage. Plotted are curves for concentrated solar power (CSP), photovoltaics (PV), wind, combined cycle gas turbine (CCGT), battery, thermal energy storage (TES), hydroelectric pumped storage (HPS), and the Kingdom of Saudi Arabia (KSA) Grid.

Figure 2 presents the optimal power generation mix for all cases using the co-optimization strategy. For the Base case, the 5.2-GW system is comprised of CSP and wind power, with CSP accounting for 75% of the total power capacity. Investments for CSP are higher than those for wind due to the availability of thermal energy storage, which allows power to be generated even when solar resources are low. There are no investments in PV solar capacity, as it is more cost-effective to invest in wind turbines than in PV, due to their slightly lower per megawatt investment costs and the more readily available renewable resource. PV would only generate power for half of the day.

Hydroelectric pumped storage systems are expensive. Their application in hot arid regions such as the Middle East would require significant maintenance to deal with high evaporation losses, further driving up costs. Given its high costs compared to other power generation technologies, the model does not invest in hydroelectric pumped storage in the Base case.

For the KSA Grid case, CSP and wind are also the only generating technologies the model selects for investment. However, the total investment capacity is lower (4.4 GW) than in the Base case, and the capacity mix is roughly 48% CSP and 52% wind. The model decides to invest slightly more in wind capacity than in CSP because of the added flexibility and reliability offered by obtaining power from the rest of Saudi Arabia. At any time, 4 GW of power can be obtained from outside of Neom. Therefore, the model can invest in more wind technology (cheaper) than in CSP because additional thermal energy storage is not required.

A summary of the capacity values for all cases is presented in Supplementary Tables 8–10. Location siting maps for the invested technologies for all cases are shown in Supplementary Figs. 5–8.

For the Wind Only and the PV Only cases, the total generating power capacities are much larger than in the Base case, and they require investments in battery storage. Because wind and solar power are intermittent resources, farm capacities must be large enough to generate power at a given hour to meet demand when the resource is available, and enough power to store in batteries to meet demand when the resource is limited. As a result, the capacity investment for the Wind Only case is 12.1 GW, which is 2.3 times larger than the Base case. However, the battery storage capacity is about the same as the thermal storage capacity required in the Base case. For the PV Only case, the power generating capacity is the largest at 40.4 GW due to the limitation of solar resources. Solar power is only available for half the day, so the system invests significantly in storage capacity and must operate the batteries more than the other cases.

Table 1 presents the power sector’s investment, operating, and total costs for all cases and strategies. As expected, the co-optimization of both sectors is beneficial in that it results in a total cost reduction for the power sector compared to the independent strategy. The cost savings are due to decreased total invested power generating capacity. Note that systems that do not invest in dispatchable power generators are significantly more expensive as they require battery capacity to supply power when renewable resources are limited or unavailable. Nevertheless, the cost savings are more significant when co-optimizing these systems with the water sector.

**Table 1:** For the power sector, all cases experience a decrease in total cost due to decreased investment spending.

Power Sector	Investment cost [M$]	Operating cost [M$]	Total cost [M$]	% Change of Total Cost
	Independent / Co-Optimization			
Base	1705 / 1626	299 / 271	2004 / 1897	−5.3
KSA Grid	1146 / 1143	359 / 348	1505 / 1490	−0.9
Wind Only	5088 / 4517	149 / 150	5236 / 4667	−10.9
PV Only	13,766 / 12,892	1094 / 1079	14,860 / 13,971	−6.0

However, systems lacking dispatchable power generators see the most considerable savings. Shown are results for the Base, Kingdom of Saudi Arabia (KSA) Grid, Wind Only, and Photovoltaics (PV) Only cases.

All co-optimized models show a decrease in total investment capacity for the power sector. Co-optimization allows for shaving peak demand by shifting necessary power consumption by the water sector to other times during the year. Such shifts reduce the total generating capacity required to meet power demand. The savings are greatest for the Wind Only and PV Only cases. For these cases, the generating capacity is significantly greater than the power demand at any given hour because these systems must generate enough electricity to meet demand and battery storage for future use.

Table 2 presents the costs incurred by the water sector for all cases under the two strategies. Unlike the power sector, which saw cost savings, the water sector experienced increased total costs, mainly due to increased investments. Note that for cases with dispatchable power generators such as the Base and KSA Grid, the percentage change in total costs between the independent and co-optimized models is negligible, 0.1% and 0.2%, respectively.

**Table 2:** For the water sector, an increase in total costs resulting from higher investment spending is particularly evident in the PV and Wind Only cases.

Water Sector	Investment cost [M$]	Operating cost [M$]	Total cost [M$]	% Change of Total Cost
	Independent / Co-Optimization			
Base	361 / 366	2347 / 2345	2708 / 2711	0.1
KSA Grid	361 / 378	2347 / 2335	2708 / 2714	0.2
Wind Only	361 / 390	2347 / 2340	2708 / 2730	0.8
PV Only	361 / 496	2347 / 2337	2708 / 2833	4.6

Shown are results for the Base, Kingdom of Saudi Arabia (KSA) Grid, Wind Only, and Photovoltaics (PV) Only cases.

For the Wind Only and PV Only cases, total costs increased by about 1% and 5%, respectively, due to increased total RO and tank storage capacity. For the Wind Only case, RO capacity increased by 8%, and storage tanks capacity increased by about 14% for the co-optimized strategy compared to the independent strategy.

The PV Only case saw a substantial increase in total desalination capacity. The co-optimized PV Only model required nearly 41% more RO desalination capacity than the independent strategy. There was also a 4% increase in water storage capacity. Because the PV system can only generate power half of the day, more desalination capacity is needed to produce excess water during hours when there is a power surplus, or when it is more cost-effective to do so (namely when batteries are not discharging to meet power demand).

Despite the increase in total costs for the water sector, the total costs of the entire system (water and power) decreased for all cases, as shown in Table 3. Co-optimization, therefore, resulted in net savings for the power-water system.

**Table 3:** For all cases, total system costs decrease when co-optimizing the power and water sectors.

System	Investment cost [M$]	Operating cost [M$]	Total cost [M$]	% Change of Total Cost
	Independent / Co-Optimization			
Base	2066 / 1993	2646 / 2615	4712 / 4608	−2.2
KSA Grid	1507 / 1521	2706 / 2683	4213 / 4204	−0.2
Wind Only	5449 / 4907	2496 / 2490	7945 / 7397	−6.9
PV Only	14,127 / 13,387	3441 / 3416	17,568 / 16,803	−4.4

Shown are results for the Base, Kingdom of Saudi Arabia (KSA) Grid, Wind Only, and Photovoltaics (PV) Only cases.

The costs of both the power and water sectors were also evaluated using levelized cost metrics, reported in Table 4. For all cases, excluding the KSA Grid case, the levelized cost of electricity (LCOE) decreased when the sectors were co-optimized. For the Base case, the reduction in LCOE is negligible, a minor 0.1% reduction. The LCOE of the independent Wind Only and PV Only cases were 134.07 USD per MWh and 47.90 USD per MWh. Co-optimizing the two sectors resulted in a 4% and 7% decrease in LCOE for the respective cases. By understanding water production patterns, capacity investment or power spillage can be decreased, which reduced the LCOE. However, in the KSA Grid case, the co-optimized model had a higher LCOE than the independent model because it buys more power from the Saudi power grid to supply water desalination facilities at more strategic times.

**Table 4:** Levelized cost of electricity (LCOE) and levelized cost of water (LCOW).

	LCOE [$/MWh]		LCOW [$/m^3^]	
	Independent / Co-Optimization	% Change	Independent / Co-Optimization	% Change
Base	17.95 / 17.93	−0.1	0.61 / 0.61	0
KSA Grid	13.34 / 13.78	3.1	0.61 / 0.54	−11.5
Wind Only	134.07 / 128.92	−3.8	0.61 / 0.61	0
PV Only	47.90 / 44.57	−7.0	0.61 / 0.61	0

Shown are results for the Base, Kingdom of Saudi Arabia (KSA) Grid, Wind Only, and Photovoltaics (PV) Only cases.

For all cases, excluding the KSA Grid, the change in LCOW is negligible.

**Fig. 3:**
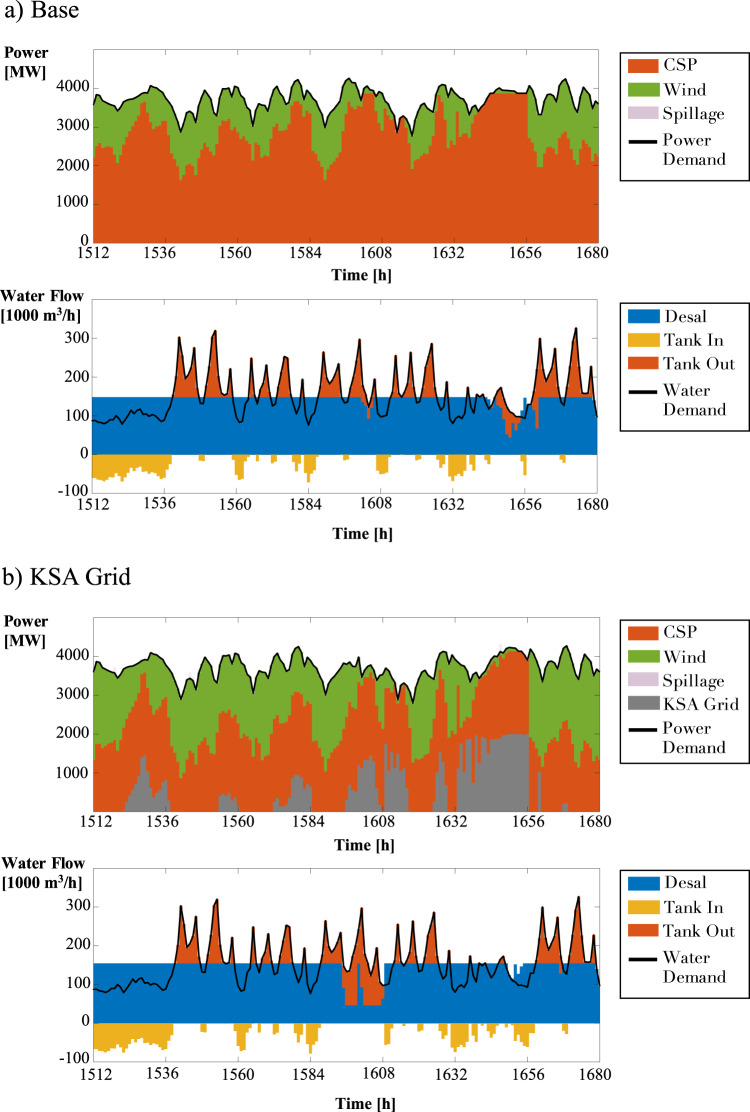
Power and water system operations in year 10 for the **a**) Base and **b**) the Kingdom of Saudi Arabia (KSA) Grid cases. Desalination plants are powered entirely by concentrated solar power (CSP) at their given node; therefore, when CSP farms operate at maximum capacity, water production decreases to meet the baseline power demand.

**Fig. 4:**
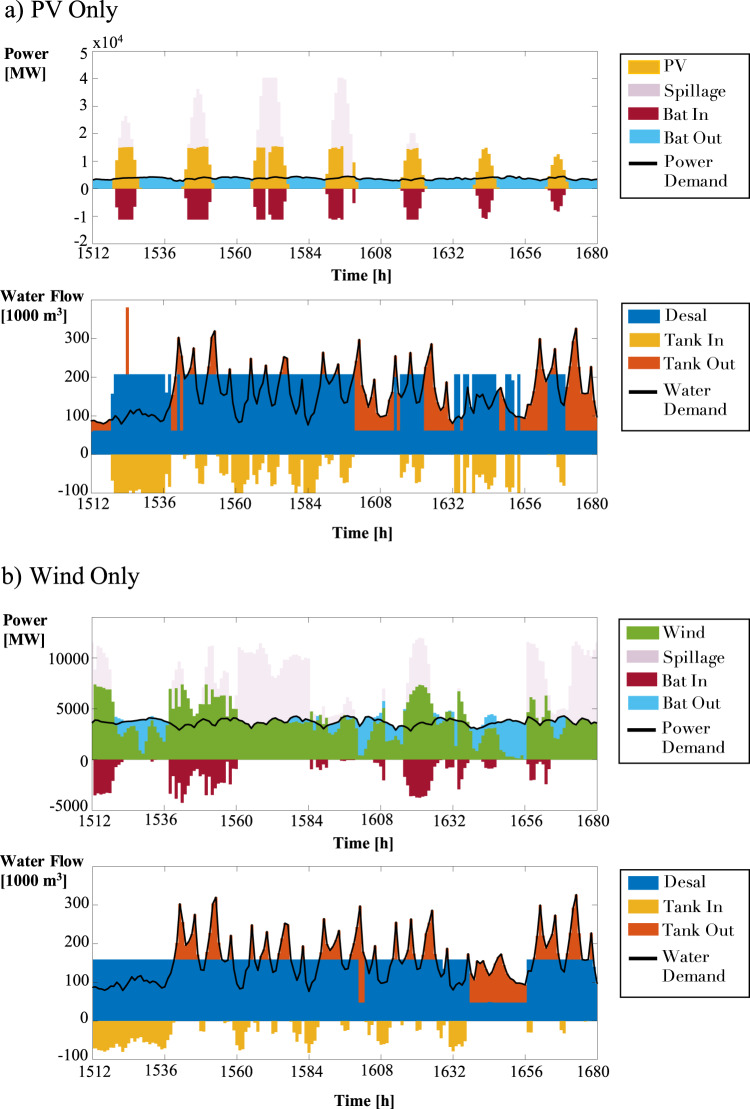
Power and water system operations in year 10 for the **a**) Photovoltaics (PV) Only and **b**) Wind Only cases. Co-optimization allows water production to decrease during hours when batteries are discharging to meet the majority of or all power demand.

The benefits of co-optimization can also be seen on the operational level, for both the power and water sectors. Figure 3a shows the operations for the Base case where water production is relatively constant until there are two dips – a minor dip between hours 1604-1607 and a more significant decrease between hours 1640-1656. Note that the reduction in water production coincides with the exact hours when CSP operates at its maximum capacity. In Fig. 4a, for the PV Only system, we see sharp drops in water production during hours when there is no sun, and the system relies solely on battery discharge to meet the total power demand. We see a similar pattern in the Wind Only case, where water production declines when batteries meet most power demand. This is seen in Fig. 4b between hours 1637 and 1656. These drops in water production are related to the need to minimize total costs. By producing less water during these periods, battery discharge is lower, and the system incurs a smaller battery operational cost.

**Fig. 5:**
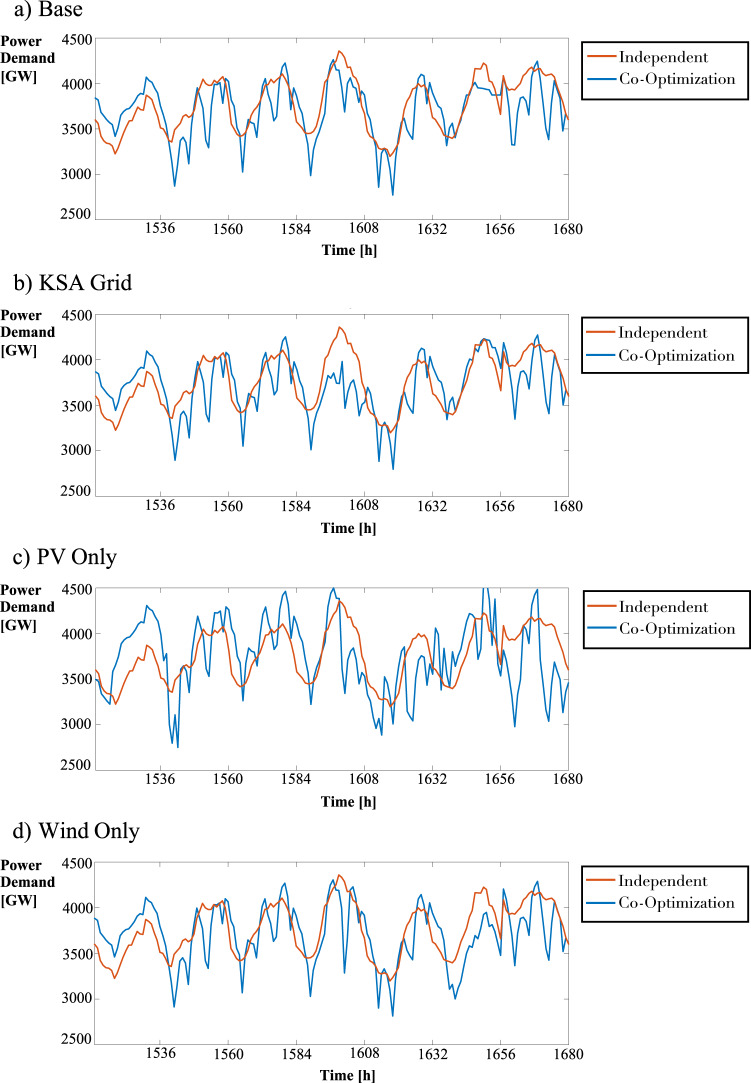
Power demand for the a) Base, b) the Kingdom of Saudi Arabia (KSA) Grid, c) Photovoltaics (PV) Only, and d) Wind Only cases under independent and co-optimization strategies. Due to co-optimization, peak shavings occur in all cases. Power consumption is shifted accordingly – at the start of the year, the co-optimized model consumes more power than the independent model. However, the co-optimized model consumes less power than the independent model at specific times throughout the remainder of the year.

From Fig. 5a, we see that the co-optimized model shaved the power demand peaks compared to the independent strategy during these times. In the case of the KSA grid, the same limiting factor occurs between hours 1596–1608 and hours 1650–1654, as depicted in Fig. 3b. It is important to note that the desalination plant is built in the same node as the CSP facility in both cases. The desalination facilities are powered by CSP exclusively; therefore, when the generators are at maximum capacity, water production decreases to ensure that the baseline power demand is met. Figure 5b shows the power consumption line from the co-optimized model falling significantly below the independent model’s consumption, evidence of power demand peak shaving.

The solutions of the proposed model are driven by the technology costs and the availability of the renewable resources (solar irradiance, temperature, and wind speed), and their hourly availability to match power and water demand. Therefore, projected technology costs impact the capacity of each technology to install, as discussed in Alraddadi et al.^19^. However, projected technology costs over the medium-term future are uncertain. In the proposed model, we do not consider uncertainty in the technology investment costs; average values are adopted. Thus, the model can be solved using a rolling horizon approach to accommodate the future variability of technology costs. The presented case study assumes a time horizon of ten years, with investments in technology capacities being made at the beginning of each year. The investments for each year are the so-called first-stage decisions that are made before the realization of the uncertain parameters. In a rolling horizon approach, the optimization problem is solved in the first iteration, and only the investments for the first year are implemented. In the second iteration, moving forward one year along the time horizon and after the first-year investments are made, technologies forecast costs are updated. The optimization problem is then solved for the next ten years. Again, only the first year (second year from the original time horizon) investments are implemented from the optimization solution. The nine years after the first year in each optimization problem prevent a limited view of future demand. In this way, technology costs are updated yearly, and their uncertainty is mitigated.

**I**ncorporating stochasticity into generation expansion planning models makes them more challenging to solve. Nevertheless, stochastic programming allows decision-makers to make decisions that are feasible over a number of scenarios and obtain a solution that is optimal for the expectation of the scenarios considered. This feasibility feature is highly relevant. For example, an optimal solution resulting from a deterministic optimization problem using average values of the uncertain parameters may be infeasible for some scenarios describing the uncertain parameters. This can occur if the optimization model does not include recourse actions (e.g., the option to obtain power from the KSA grid) to handle some investment decisions. Besides, stochastic programming problems can unveil solutions that are not available by optimizing one scenario at a time.

To assess the quality of the stochastic programming solutions, we present the values of the stochastic solution (VSS) obtained. We also report the expected value of perfect information (EVPI), which indicates the maximum price that decision-makers should pay to obtain perfect information. Table 5 presents the VSS and EVPI values for the four cases presented. On average, decision-makers can save 115 million USD for the Base case by considering uncertainty. The VSS is also greater than zero for the remaining cases, indicating potential cost savings when using a stochastic programming approach. The EVPI results show that perfect information is less important in the KSA Grid case, which results from the constant power availability provided by the KSA grid, and more relevant in the PV Only case.

**Table 5:** Value of Stochastic Solution (VSS) and Expected Value of Information (EVPI).

	VSS [M$]	EVPI [M$]
Base	115	423
KSA Grid	85	195
Wind Only	111	1195
PV Only	60	3391

Shown are results for the Base, Kingdom of Saudi Arabia (KSA) Grid, Wind Only, and Photovoltaics (PV) Only cases.

In the multiple cases addressed in this work, representative days are adopted in the stochastic programming model to overcome the computational burden of considering a full-time resolution with 8760 h per year in a multi-year horizon model. This reduces the model complexity and computational burden by reducing the model size in the time dimension. However, an approximation is introduced by compressing the variability of the renewable resources time series into a number of representative days. To investigate the trade-offs of using representative days, we compare results from models using representative days and a full-time resolution in Supplementary Note 9. The results show that a stochastic planning model using a multi-year horizon with a full-time resolution is computationally intractable, highlighting the importance of using representative days in these types of models. Considering a single-year horizon stochastic model, the results with both time representations lead to similar investment decisions in various technologies. However, compared to the representative day model, the full-time resolution model shows a 5.12% and 2.21% increase in CSP and wind capacities, respectively, with a 193.5% increase in computing time. The results of the deterministic model with both approaches follow a similar trend, with equivalent investments in various technologies. However, the full-time resolution model shows a 3.00% and 1.64% increase in CSP and wind capacities, respectively, with a 167% increase in computing time. Overall, these results highlight the computational advantage of using representative days for multi-year models. Furthermore, close investment decisions are obtained using representative days compared with the full-time resolution models. These results are summarized in Supplementary Tables 18 and 19.

The impact of the water sector on the power grid is significant as co-optimizing the two systems reveals changes in the investment strategy and shifts in the power demand. Thus, co-optimization over multiple sectors can be instrumental for decision-makers to make more effective decisions regarding operations and system investments. The results show that co-optimizing the power and water sectors results in lower total costs. In particular, if only renewable energy sources are selected, the total cost reduction is more significant. The operational results also demonstrate that the water desalination operations adapt to the availability of wind and solar resources, which prevents the model from making further investments. Other sectors of importance would include the transportation sector, where there is a push towards utilizing electric or hydrogen-fueled cars and the chemical sector. Neom is constructing the largest hydrogen facility to produce hydrogen using renewable energy sources and water. Hydrogen production will directly impact Neom’s energy storage capability and power demand. In the same realm, the electrification of the transportation fleet will also drive power demand and serve as a distributed battery system in Neom for short-term energy storage.

The Base case results provide the optimal generation mix for a fully renewable power system. However, we also analyzed one case where Neom could obtain power from the KSA grid based on fossil fuels. The carbon emissions related to this power generation can be accounted for, and a mechanism to offset emissions can be implemented. The hydrogen produced can be utilized to produce e-fuels, carbon-based fuels such as methanol, and formic acid^9^. The production of these fuels will also require carbon dioxide to be sequestered from the air or from industry. In a similar context, geothermal technology that uses carbon dioxide as the injection fluid can generate power while reducing the total emissions of the system.

Future work will evolve in two directions: 1) quantifying carbon emissions and addressing a dynamic carbon offsetting system; and 2) coupling additional sectors (hydrogen production, transportation, e-fuels) in our co-optimization model to highlight sector interactions and the collective and individual reliability of the systems.

The proposed model was applied to Neom, Saudi Arabia; however, it is a generic model which can be adapted to other regions given the respective data inputs. The results obtained for Neom may provide insights into other systems with similar climates, renewable resource availability, or a need for desalination. These regions include the Southwestern region of the United States, parts of Australia, Chile, South Africa, and the MENA region^28^.

## Methods

### Stochastic programming model

We developed a stochastic programming model to co-optimize the expansion planning of water desalination, power generation, and transmission lines. The model considers long-term uncertainty for electricity and water demand and variability of the availability of renewable resources. The decision framework involves a two-stage decision process encapsulated into a single model. The first-stage decisions are investment decisions at the start of each year to install power generation facilities, storage technologies, transmission lines, and water desalination systems. The second-stage decisions are related to power generation operations, water desalination operations, and storage inputs and outputs. The model equations can be found in Supplementary Note 6.

The objective function consists of the total investment cost plus the expected operating cost from 2020 to 2029. To minimize the objective function, we approximate a multiple-stage decision process with a two-stage framework. Investment decisions for each year are all treated as first-stage decisions. The model should be run every year to improve the approximation, using updated demand growth forecasts to determine the investment decisions.

Power generating and storage technologies include concentrated solar power (tower), photovoltaics, hydroelectric pumped storage, combined cycle gas turbines, and batteries. Water desalination and storage technologies include reverse osmosis desalination and water storage tanks. Investment decisions are based on the investment costs of the technologies themselves in a given year. We assume a 1% decrease in technology investment costs every year. The demand growth uncertainty is revealed in the subsequent stage, and operating decisions are made regarding power generation, water desalination, and storage inputs and outputs. These decisions are made considering the variable operating costs of the technologies and the resource availability of renewables. Operating decisions are made on an hourly scale.

The uncertainty in interannual demand growth of both water and electricity is represented by three scenarios: low, medium, and high demands, each characterized by a corresponding probability of occurrence. We assume that water demand growth is related to electricity demand growth; a high demand scenario means that both water and electricity demands are high. A low-demand scenario means that both water and electricity demands are low.

Electricity demand, water demand, and renewable resources available for wind and solar power generation vary throughout a given day and year. We use representative days rather than all 365 days (8760 h) of a year to make the model tractable.

The model is formulated as a mixed-integer linear program using the modeling system GAMS^29^ 27.2 and solved with a branch & bound algorithm within the CPLEX 12.8^30^ package using an optimality gap tolerance of 0.1% and a time limit of 168 hours. All optimization runs were performed on the KAUST IBEX computer cluster using exclusive nodes, each having 40 Intel Gold 6148 @ 2.6-GHz processors and 384 GB of RAM.

### Electricity and water demand profiles

Because Neom is a planned project, there is no historical electricity or water demand data. Therefore, we use annual projections between 2020 through 2029 for both water and electricity (all sectors) demand. The scenarios are dependent on the projected population sizes. Because we are co-optimizing the electricity and water systems, we subtract the electricity demand from desalination based on water demand projections using a conversion factor of 4 kWh/m^3^. The model accounts for the power consumption needed for desalination when it determines how much water to desalinate.

We derived the yearly water and electricity demand profiles for Neom from the demands at KAUST - an academic institution of 7000+ residents approximately in Thuwal, Saudi Arabia. We obtained the 2018 hourly water and electricity demand curves for KAUST. We applied the profiles to Neom by normalizing the area under the curve to 1 and multiplying it by the total annual demand for Neom in a given year.

### Climate data

We use direct normal irradiance, global horizontal irradiance, and temperature data for Jan 2008 through June 2019 (11.5 years) at a 5 km resolution spanning the entire region of Neom. This data was obtained from a high-resolution evaluation of the solar energy resources over the Arabian Peninsula using reanalysis data generated by the Weather Research and Forecasting (WRF) Solar model. Reanalysis data was validated using daily observations at 46 in-situ radiometer stations throughout Saudi Arabia^31^.

Wind speed data for the Neom region over the same timespans and resolution as the irradiance and temperature data was used. The wind speed data was derived from a high-resolution reanalysis generated using WRF and validated with data from buoys, scatterometers, and altimeters placed throughout the area^32^.

### *k-*means clustering for representative regions and days

Clustering methods involve several steps, (1) data normalization, (2) data assignment, and (3) cluster representation^33^. We normalize our data using the z-scoring full scope normalization on each attribute to shift the mean to 0 and standard deviation to 1. In the data assignment step, we use a partitional clustering method known as *k-*means clustering, which uses Euclidean distance as its distance measure and the centroid (mean) as its cluster center. Once the clusters are determined, each cluster is represented by one of its assigned observations, typically done by selecting the observation that minimizes its distance to the cluster center.

The goal of *k*-means clustering is to select a set of *k* clusters that minimizes the sum of the squared distance between the rows of a dataset and the cluster centroid they are assigned to; this sum is called the potential. The *k*-means approach most commonly used is Lloyd’s algorithm^34^, which begins by selecting *k* arbitrary centroids, chosen uniformly at random from the normalized dataset. Each row is assigned to the nearest centroid, determined by calculating the Euclidean distance between a given row to the *k* possible centroids. This step forms the initial clusters. The centroid is then recalculated for each cluster by taking the mean of all data points in the cluster. We used an alternative approach, *k*-means + +, which initializes Lloyd’s algorithm with random starting centers with specific probabilities proportional to their contribution to the overall potential^35^. Clustering was performed on MATLAB R2020a using the *kmeans* function. For more information on *k*-means + +, see Supplementary Note 2.

### Representative regions

Clustering methods are used to divide Neom into regions according to climate conditions (e.g., wind speed, solar irradiance, and temperature)^10^. Climate data is available for a spatial resolution of 5 km, accounting for 4270 locations within Neom. *k*-means clustering is used to group these locations into ten representative regions. More information can be found in Supplementary Note 3.

### Representative days

Clustering methods are also used to capture the hourly variability from renewable generation and power and water demand^33,36^. This approach decreases the model size by aggregating long time series into shorter time slices. We use *k*-means clusters to group 4194 days (~11.5 years) into seven representative days per year. The days were clustered based on the water and power demand, wind and solar availability, and temperature. More information can be found in Supplementary Note 4.

### Capacity factors of renewable technologies

Global Horizontal Irradiance (GHI), the total amount of radiation received by a horizontal surface, and temperature data were used to obtain the capacity factors of photovoltaic panels based on a standard 0.25 kW solar cell. Direct Normal Irradiance (DNI), the amount of radiation received per unit area by a surface perpendicular to the incoming rays, is used to obtain power outputs for CSP farms. Wind power output was obtained from wind speed data by applying the power curve of Vestas 150-4.2^37^, a 4.2 MW wind turbine with a hub height of 150 m. Supplementary Note 5 presents the equations to obtain the power production and capacity factors.

### Water desalination

Several technologies are commonly utilized for water desalination, such as multi-effect distillation (MED), multistage flash (MSF) distillation, and reverse osmosis (RO). Our model includes only RO and limits the operational constraints, such as ramping rates, to reduce computational complexity. We assume that a RO plant cannot be shut down as periodic shutdowns generally damage the permeable membranes. Electricity consumption for a typical RO plant is held constant throughout the project lifetime at 4 kWh/m^3^.

### Preoptimization calculation of annualized costs

Annualized cost is a metric that allows decision-makers to compare the cost-effectiveness of technologies with different lifespans, accounting for a given annuity factor. The definitions are given in Supplementary Note 1. Annualized cost values are given in Supplementary Tables 1 and 2.

### Postoptimization analysis using levelized costs of electricity and water

Levelized costs of electricity (LCOE) and water (LCOW) metrics are used to compare the competitive costs of different investment plans. They describe the average net present value of electricity generation or water production over the lifetime of the project. Their definitions are given in Supplementary Note 7.

### The value of the stochastic solution and the expected value of perfect information^38^

The value of the stochastic solution (VSS) is a measure that quantifies the cost of planning without considering uncertainty. The expected value of perfect information (EVPI) is the maximum amount one should pay for complete and accurate information about the future. The definitions of both terms are given in Supplementary Note 8.

### Reporting summary

Further information on research design is available in the Nature Research Reporting Summary linked to this article.

## Supplementary information

Supplementary Information

Description of Additional Supplementary Information

Supplementary Data 1

Supplementary Data 2

Reporting Summary

## Data Availability

The data relating to the representative days and regions, capacity factors, and electricity and water demand are available in a Supplementary Data file: Supplementary_Data_2.xlsx. For original climate data, please contact the authors in^31^ and^32^. The cost information and performance parameters for technologies and transmission lines are available in the Supplementary Information. The raw data for Figs. 2–5 are available in a Supplementary Data file: Supplementary_Data_1.xlsx.

## Code availability

This work did not use a specific developed algorithm to perform the optimizations. The mathematical programming models were written in the modeling system GAMS 27.2 and solved with the commercial solver CPLEX 12.8. Detailed descriptions of the sets, parameters, objective function, constraints, and variables are available in the supplementary information document. The clustering analysis applied to the climate data was performed using the commercial software MATLAB R2020a.
