# Supplementary material for: Simulated co-optimization of renewable energy and desalination systems in Neom, Saudi Arabia
Source: Nat Commun. 2022 Jun 18;13:3514. doi: 10.1038/s41467-022-31233-3 (PMC9206678; doi:10.1038/s41467-022-31233-3)
Supplement: Supplementary file 1 — Supplementary Information [file 41467_2022_31233_MOESM1_ESM.pdf]

Supplementary Information

Simulated co-optimization of renewable energy and desalination systems in Neom, Saudi Arabia

Jefferson A. Riera[1], Ricardo M. Lima[2], Ibrahim Hoteit[1], Omar Knio[2*]

[1]Physical Science and Engineering Division, King Abdullah University of Science and Technology, (KAUST), Thuwal 23955-6900, Saudi Arabia. [2]Computer, Electrical and Mathematical Sciences & Engineering Division, King Abdullah University of Science and Technology, (KAUST), Thuwal 23955-6900, Saudi Arabia. [*]email: omar.knio@kaust.edu.sa

Supplementary Figures.

a)

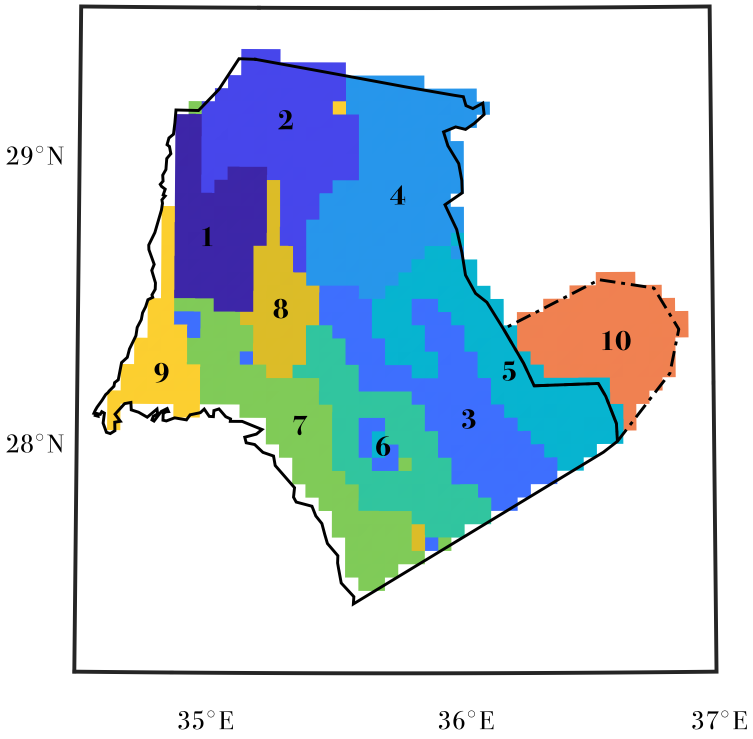

b)

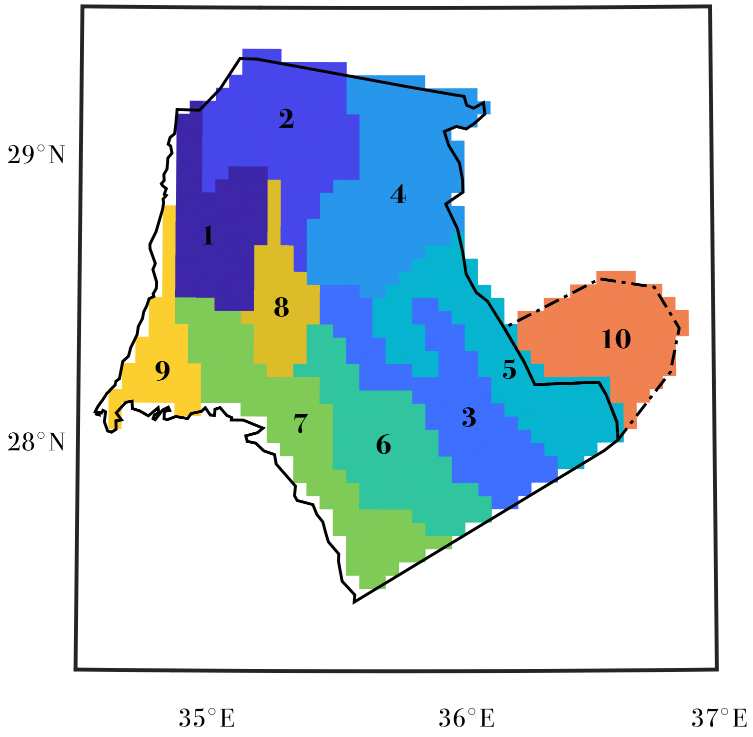

Supplementary Figure 1: (a) Clusters after spatial clustering assignments; (b) Clusters after manual reassignment.

a)

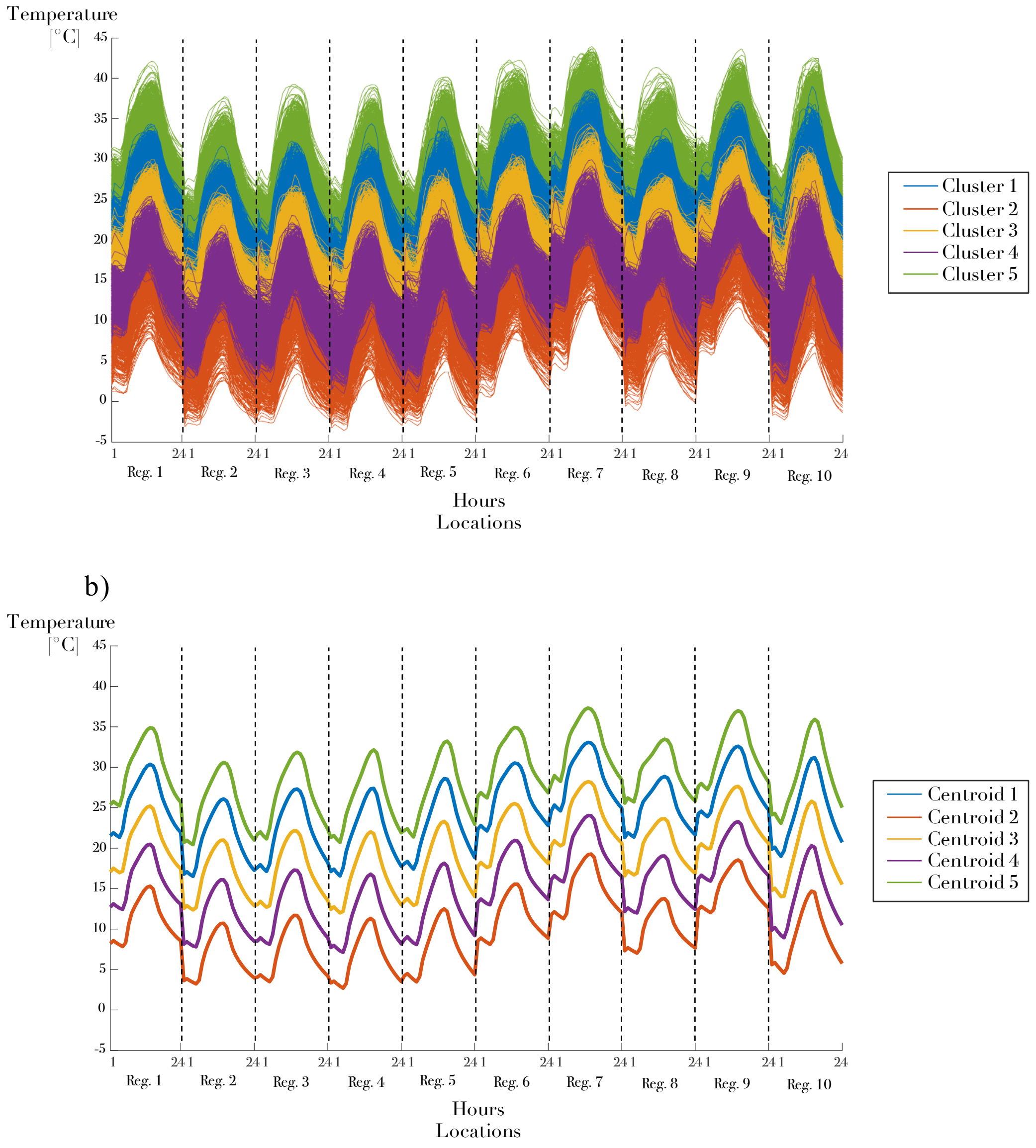

Supplementary Figure 2: (a) Temporal *k*-means clustering assigns all 4194 days to 5 different clusters. (b) Centroids are determined by taking the mean of in-cluster days.

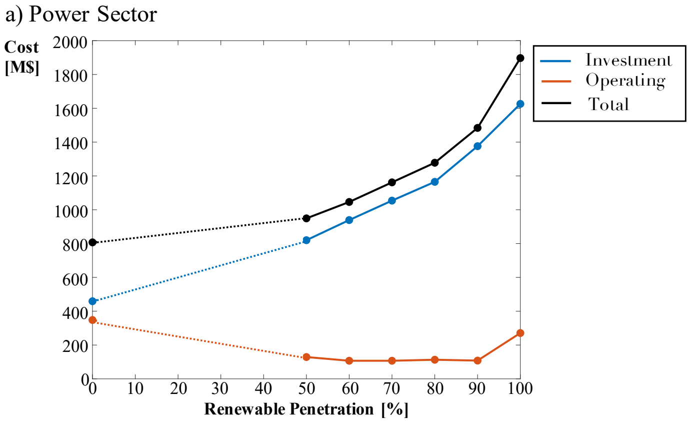

Supplementary Figure 3: Costs dependent on renewable penetration when desalination consume 20% of total power. (a) Power sector costs as a function of renewable penetration. With increasing levels of renewable penetration, investment costs increase. Operating cost decreases, on the other hand decrease at first and then begin to increase when the system relies on more concentrated solar power. (b) Water sector costs as a function of renewable penetration. Water sector is relatively unaffected by increasing renewable penetration in the power system.

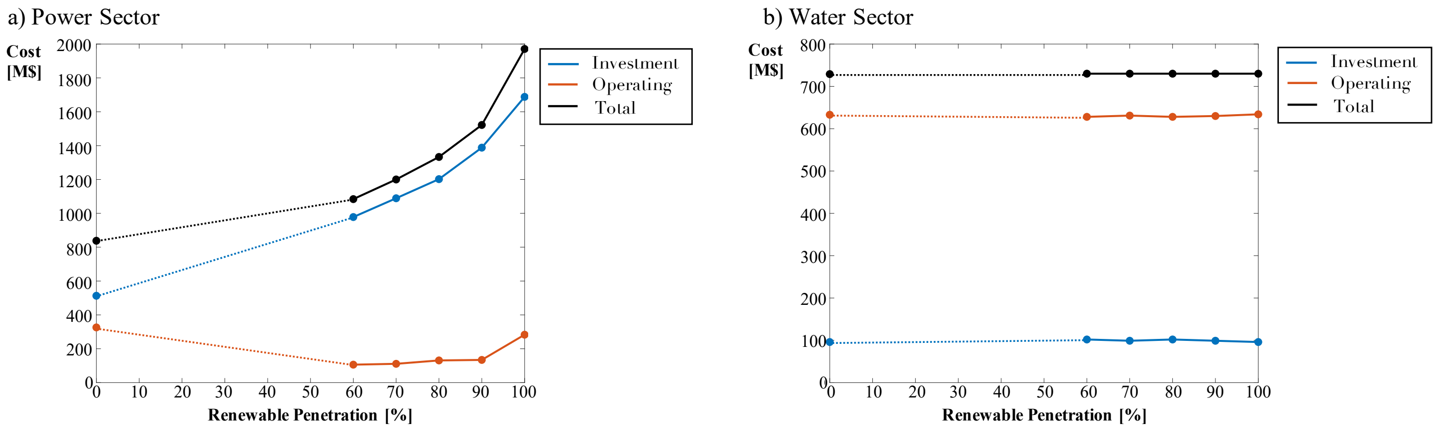

Supplementary Figure 4: Costs dependent on renewable penetration when desalination consume 4% of total power. Results are similar to the 20% results in Supplementary Figure 3.

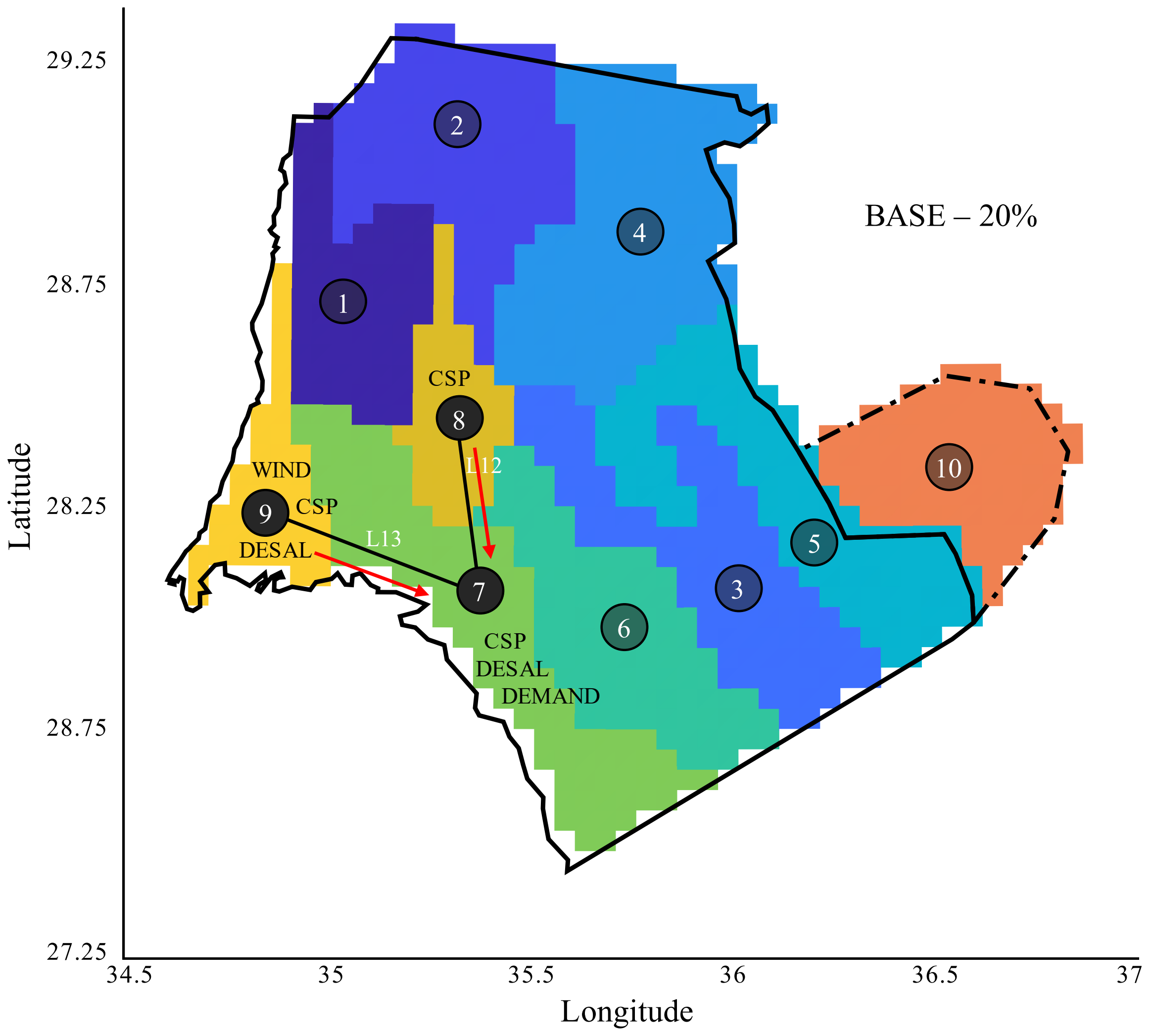

Supplementary Figure 5: Transmission network and net power flows for Base case for 20% power consumption from desalination. The system depends heavily on concentrated solar power (CSP) to meet power demand. Desalination facility at node 9 relies entirely on power generated at that node.

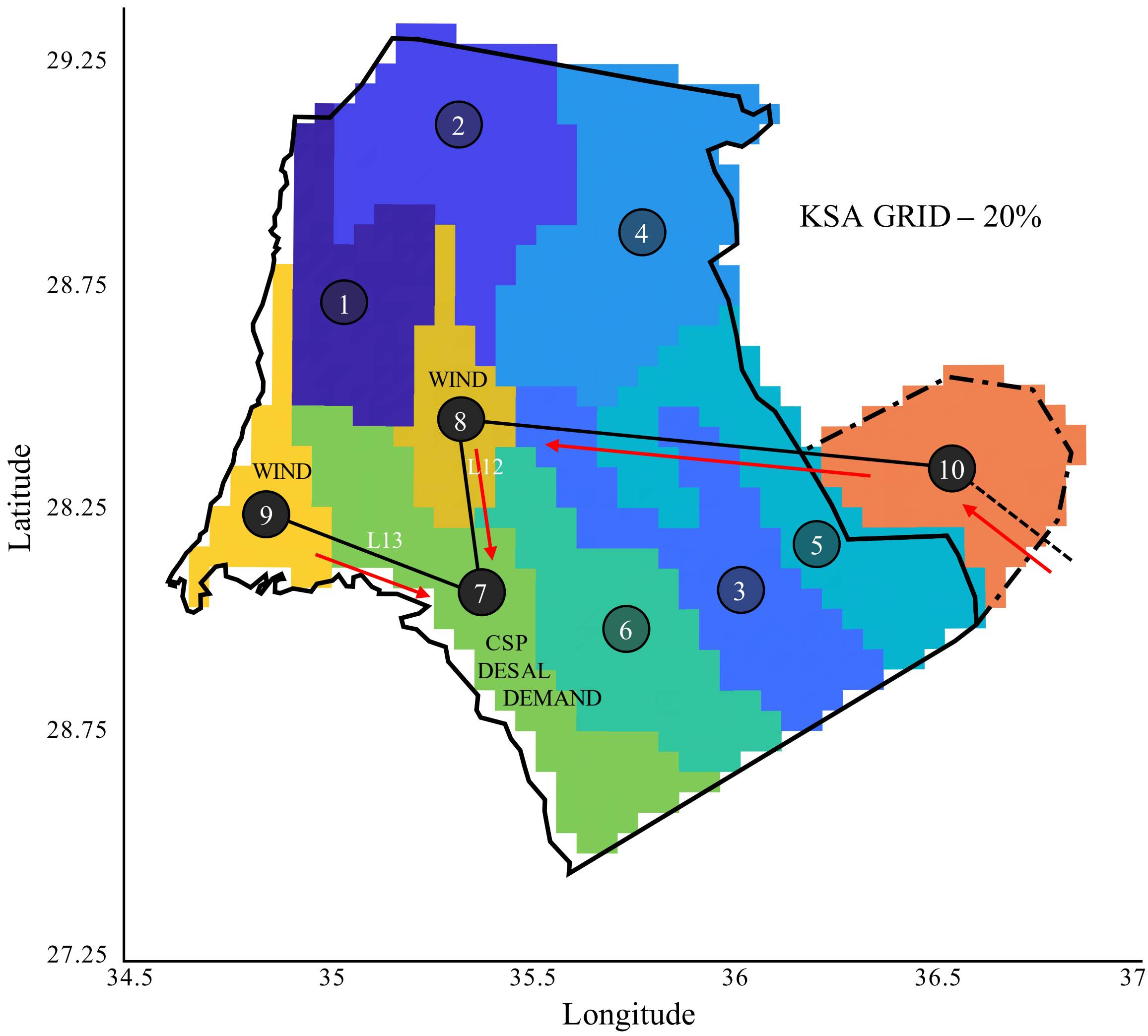

Supplementary Figure 6: Transmission network and net power flows for the Kingdom of Saudi Arabia (KSA) Grid case for 20% power consumption from desalination. Transmission lines are built to connect with the KSA grid to obtain power. Wind plants are installed in regions 8 and 9, whereas concentrated solar power (CSP) and desalination facilities are located in region 7.

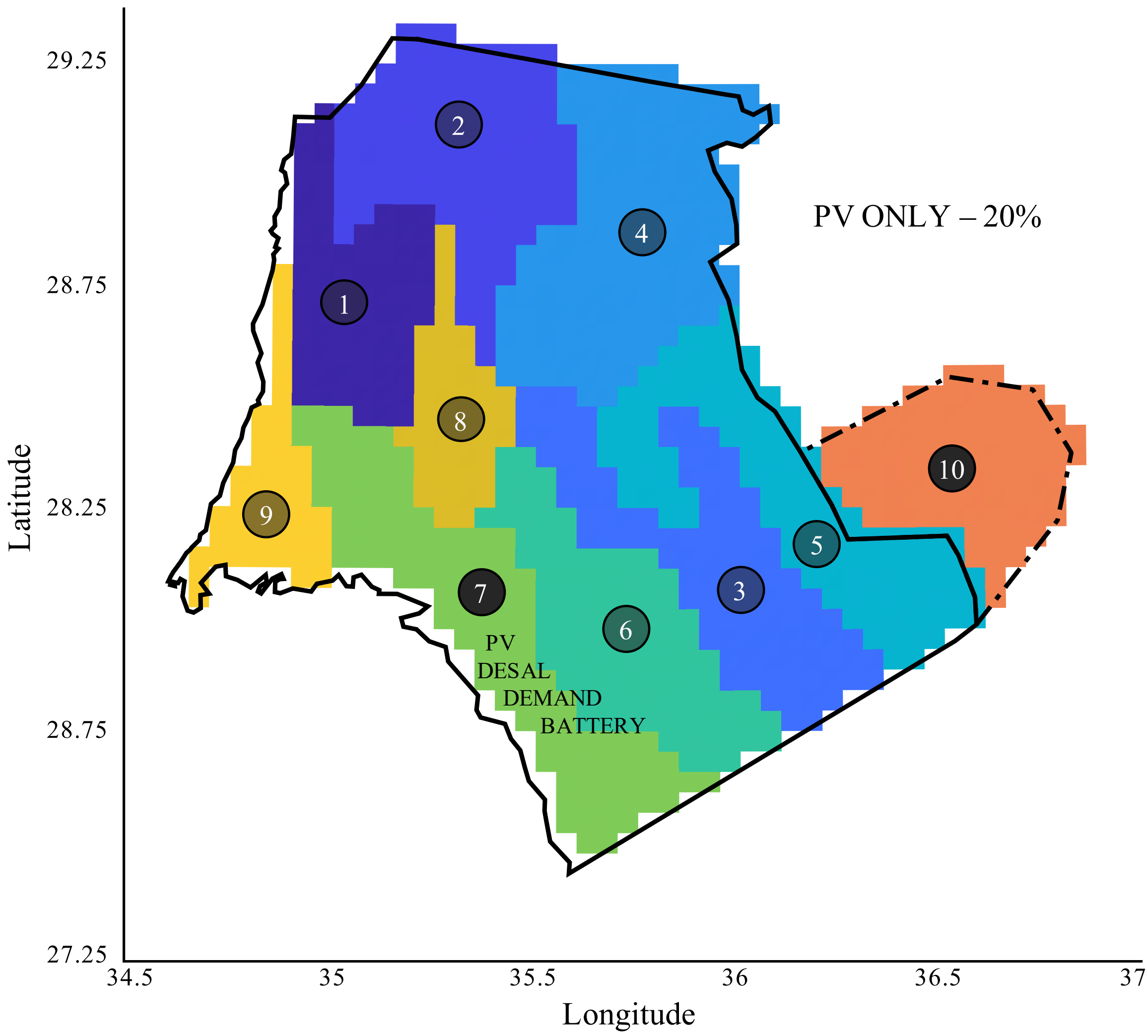

Supplementary Figure 7: Transmission network and net power flows for photovoltaics (PV) Only case for 20% power consumption from desalination. Solar availability is relatively constant throughout Neom; therefore, building a large enough PV farm in node 7, where demand is located, is most cost effective.

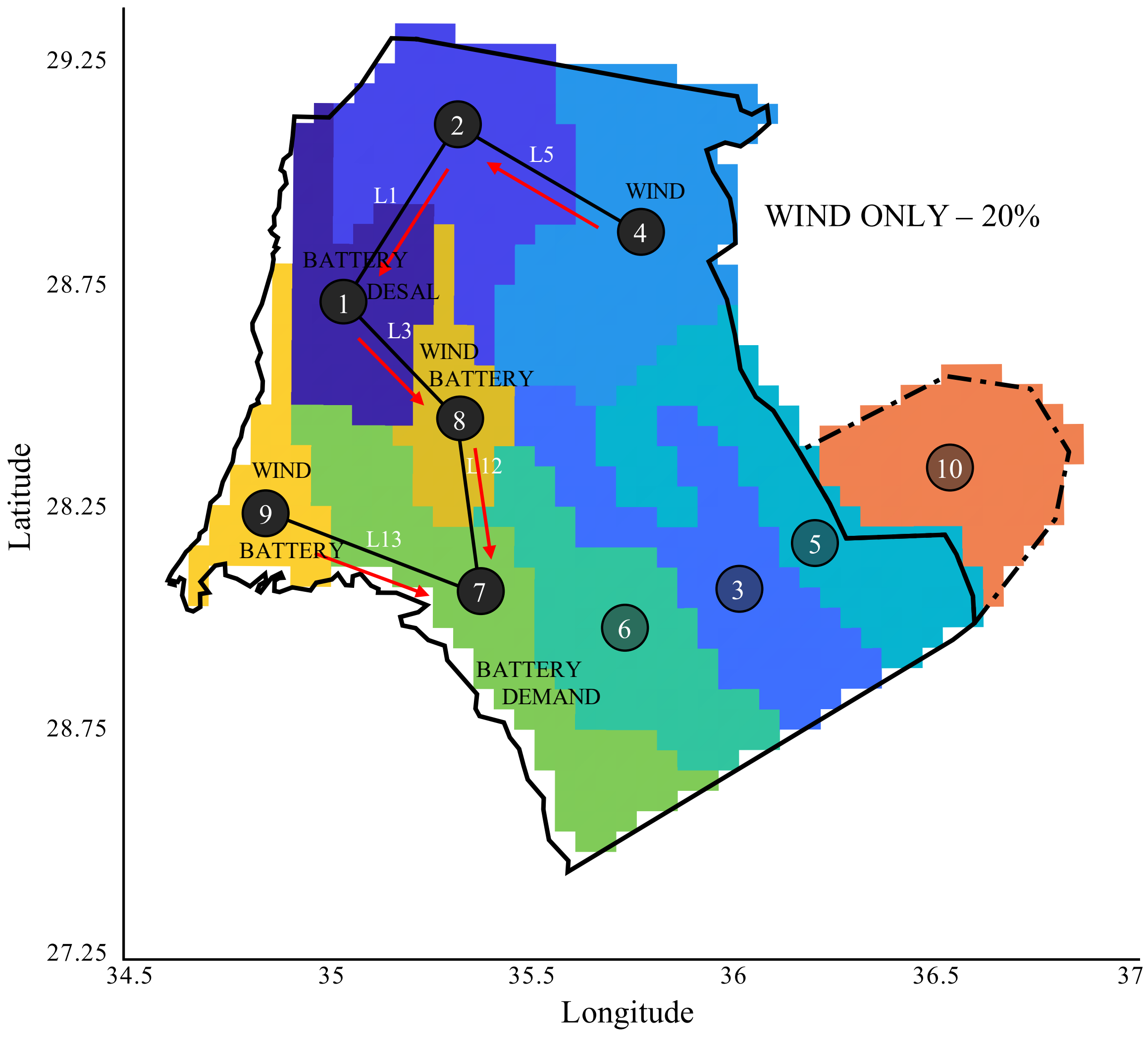

Supplementary Figure 8: Transmission network and net power flows for the Wind Only case with 20% power consumption from desalination. Large wind resources in nodes 4, 8 and 9 are coupled with batteries to supply sufficient power to meet desalination power demand and baseline power demand.

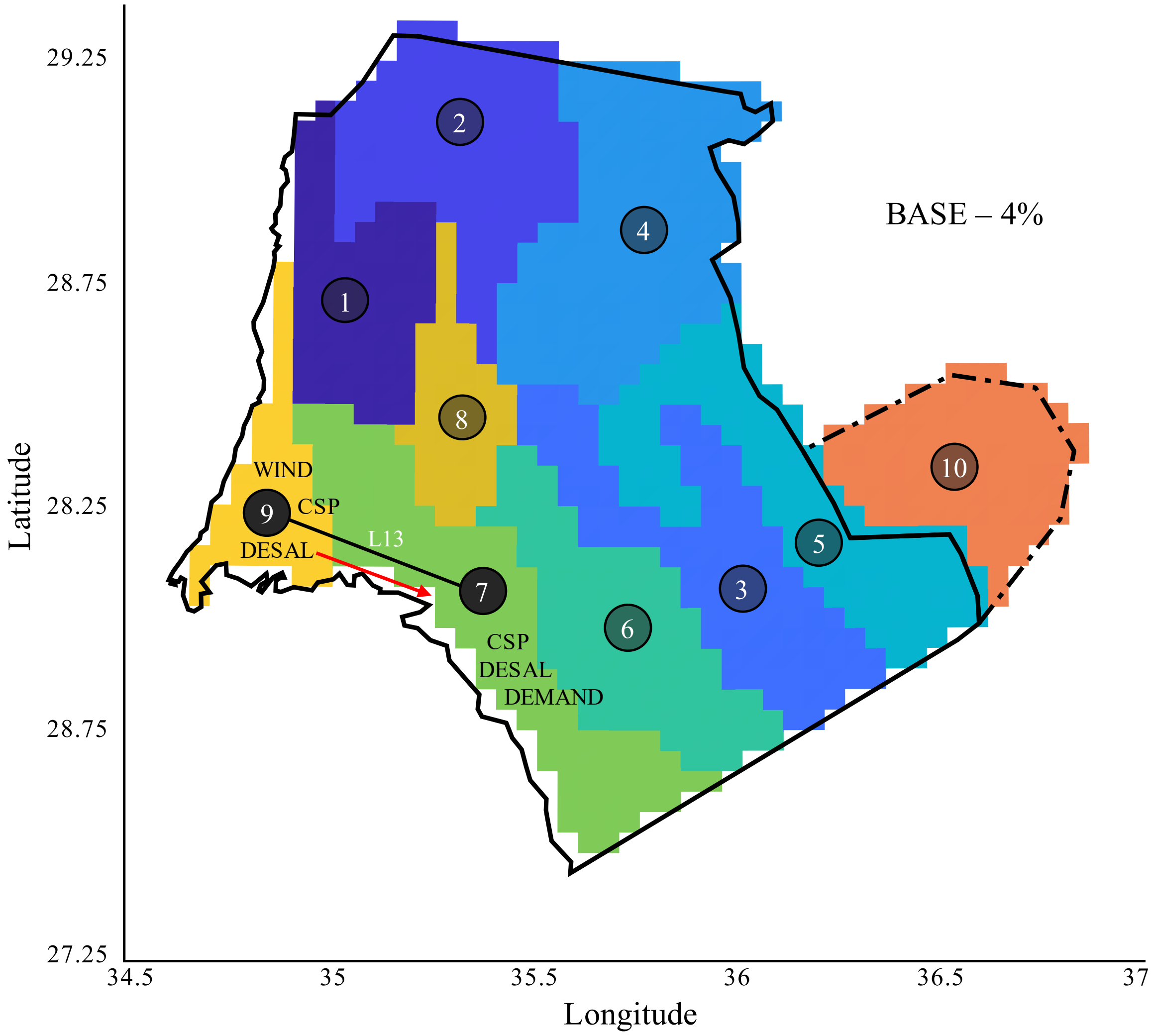

Supplementary Figure 9: Transmission network and net power flows for Base case for 4% power consumption from desalination. A two-node system where desalination demand is met by entirely by renewable power. Only wind and concentrated solar power (CSP) technologies are built to meet power demand.

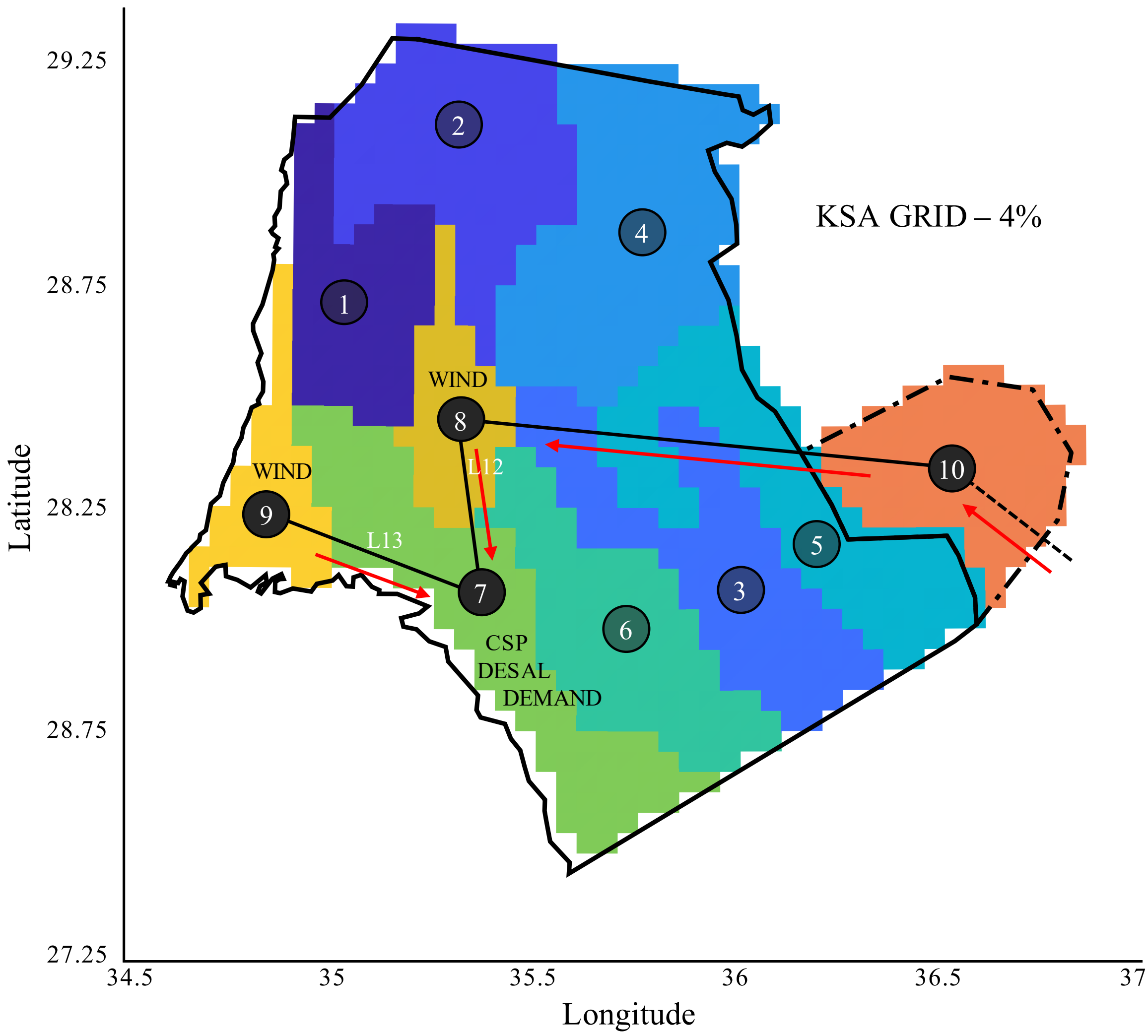

Supplementary Figure 10: Transmission network and net power flows for the Kingdom of Saudi Arabia (KSA) Grid case for 4% power consumption from desalination. Transmission lines connect Neom to the KSA grid at node 10, in order to meet power demand. Wind facilities are located in nodes 8 and 9, whereas concentrated solar power (CSP) and desalination plants are located in node 7.

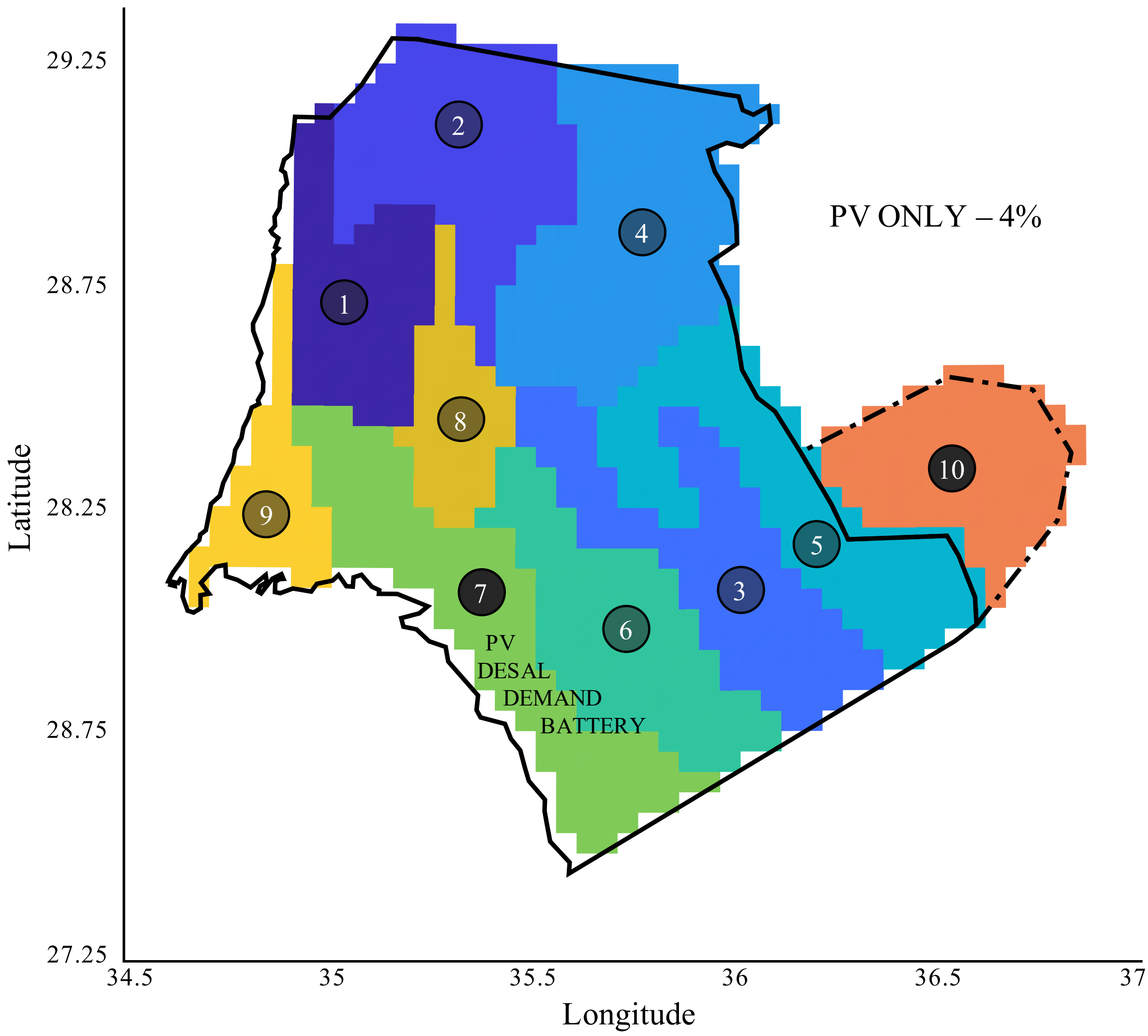

Supplementary Figure 11: Transmission network and net power flows for the photovoltaics (PV) Only case for 4% power consumption from desalination. Solar power resources are constant throughout Neom, so an isolated system is created at node 7, where there is power demand.

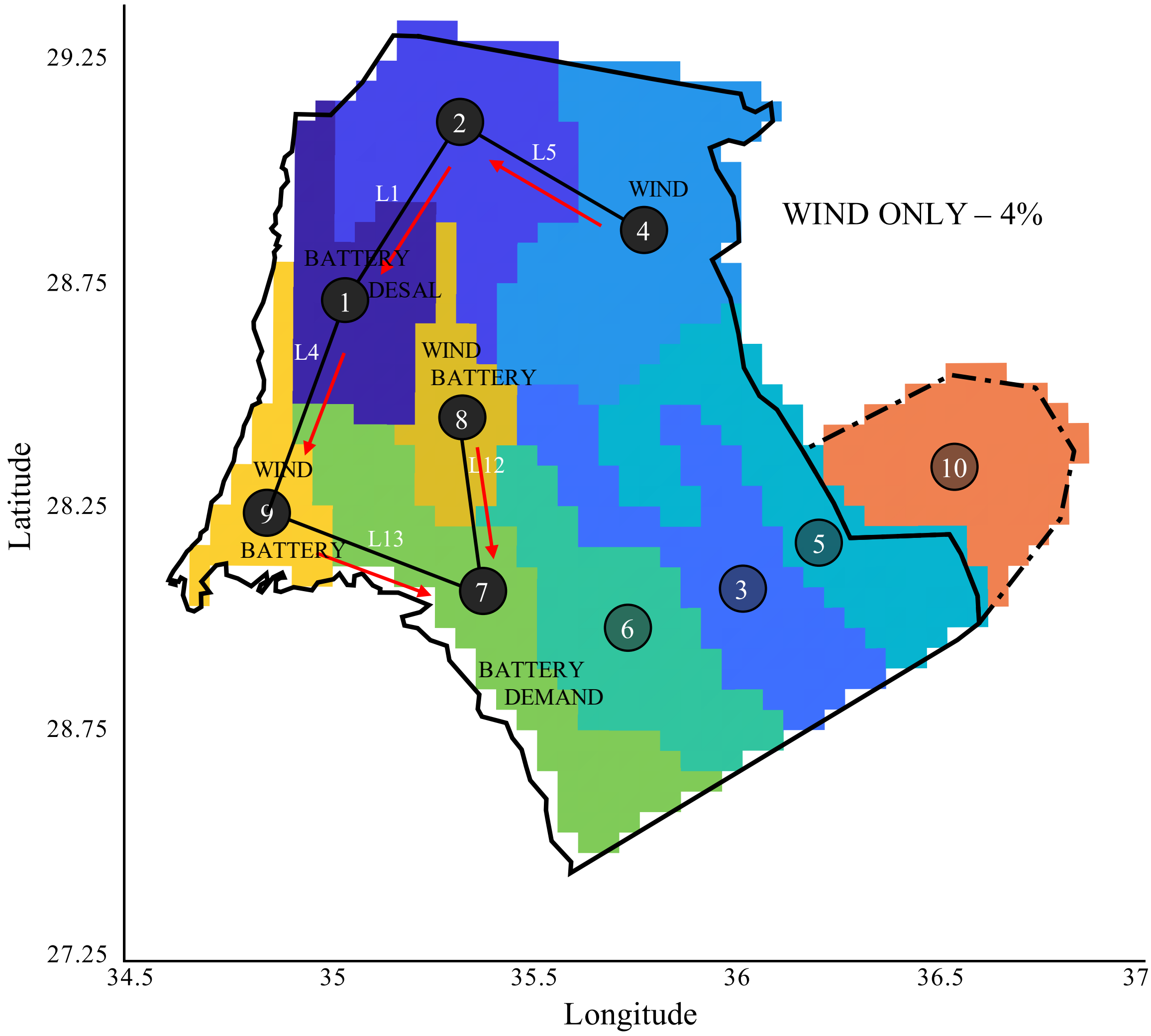

Supplementary Figure 12: Transmission network and net power flows for the Wind Only case with 4% power consumption from desalination. Good wind resources are scattered throughout the kingdom. Wind farms are built at node 4 far away from the baseline demand. Desalination plants in node 1 are powered by winds farms and battery storage.

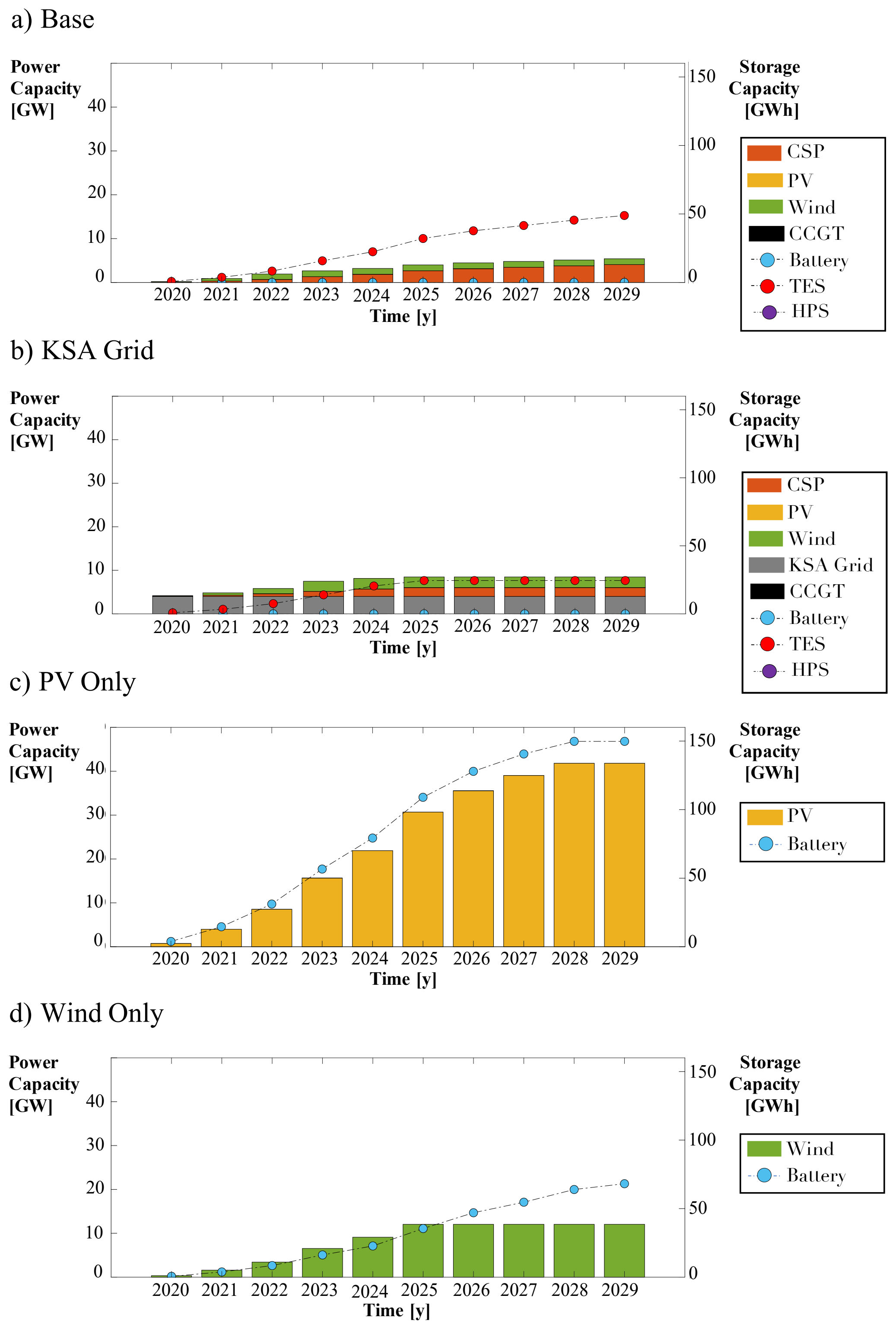

Supplementary Figure 13: Power generating capacities for all cases when desalination accounts for only 4% of total power consumption. Plotted are curves for concentrated solar power (CSP), photovoltaics (PV), wind, combined cycle gas turbine (CCGT), battery, thermal energy storage (TES), hydroelectric pump storage (HPS), and Kingdom of Saudi Arabia (KSA) grid.

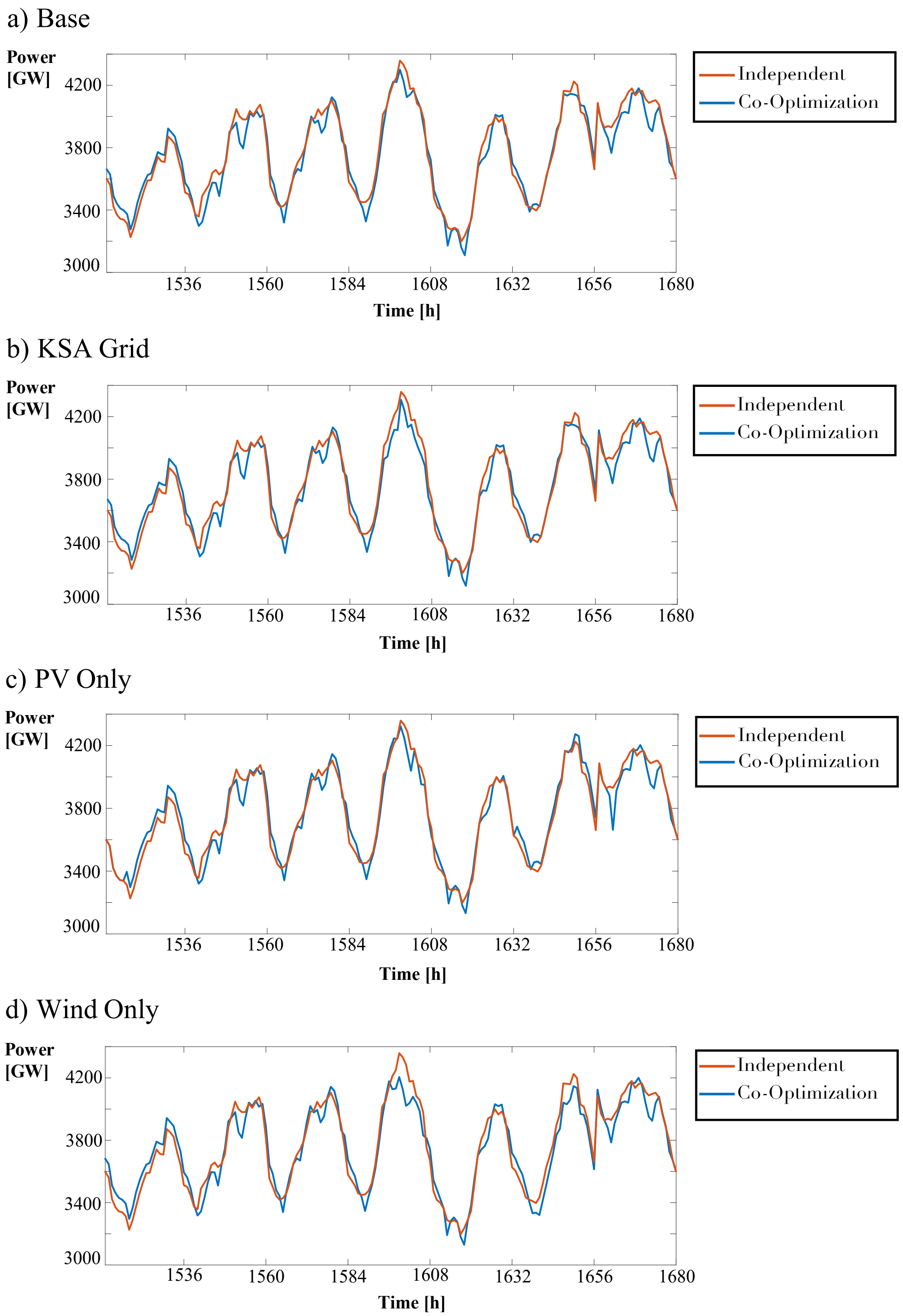

Supplementary Figure 14: Power consumption for the Base, Kingdom of Saudi Arabia (KSA) Grid, Photovoltaics (PV) Only, and Wind Only cases. Desalination accounts for 4% total power consumption. In all cases the peak power consumed is lower than in the co-optimized strategy. The Wind Only case shows the largest peak reduction during the summer months compared to all other cases.

Supplementary Tables

Supplementary Table 1: Investment and Operating Cost of Power Generating and Storage Technologies. Provided are estimates for combined cycle gas turbine (CCGT), concentrated solar power (CSP), photovoltaics (PV), wind, battery, and hydroelectric pump storage (HPS) technologies.

Technology	Costs	2020	2021	2022	2023	2024	2025	2026	2027	2028	2029
CCGT	AIC [$/kW]	97.93	96.95	95.99	95.03	94.08	93.13	92.2	91.28	90.37	89.46
	VOC [$/MWh]	3.61	3.61	3.61	3.61	3.61	3.61	3.61	3.61	3.61	3.61
CSP	AIC [$/kW]	381.16	377.35	373.57	369.84	366.14	362.48	358.85	355.26	351.71	348.19
	VOC [$/MWh]	3.5	3.5	3.5	3.5	3.5	3.5	3.5	3.5	3.5	3.5
PV	AIC [$/kW]	167.03	165.36	163.71	162.07	160.45	158.84	157.25	155.68	154.13	152.58
	VOC [$/MWh]	0	0	0	0	0	0	0	0	0	0
Wind	AIC [$/kW]	159.2	157.61	156.04	154.48	152.93	151.4	149.89	148.39	146.9	145.44
	VOC [$/MWh]	0	0	0	0	0	0	0	0	0	0
Battery	AIC [$/kW]	47.69	47.21	46.74	46.27	45.81	45.35	44.9	44.45	44.01	43.57
	VOC [$/MWh]	7.26	7.26	7.26	7.26	7.26	7.26	7.26	7.26	7.26	7.26
HPS	AIC [$/kW]	453.37	448.84	444.35	439.91	435.51	431.15	426.84	422.57	418.35	414.16
	VOC [$/MWh]	0.51	0.51	0.51	0.51	0.51	0.51	0.51	0.51	0.51	0.51

AIC: annualized investment cost; VOC: variable operating cost

Supplementary Table 2: Investment and Operating Costs of Water System Technologies. Provided are estimates for reverse osmosis (RO) and tank storage technologies.

Technology	Costs	2020	2021	2022	2023	2024	2025	2026	2027	2028	2029
RO	AIC [$/($m^3$/hr)]	2396.68	2372.71	2348.98	2325.49	2302.24	2279.22	2256.42	2233.86	2211.52	2189.41
	VOC [$/$m^3$]	0.5	0.5	0.5	0.5	0.5	0.5	0.5	0.5	0.5	0.5
Tank Storage	AIC [$/($m^3$/hr)]	6.4	6.33	6.27	6.21	6.14	6.08	6.02	5.96	5.9	5.84
	VOC [$/$m^3$]	0.02	0.02	0.02	0.02	0.02	0.02	0.02	0.02	0.02	0.02

AIC: annualized investment cost; VOC: variable operating cost

Supplementary Table 3: Transmission Line Technical Parameters

Line	Corridor	Length [km]
l_1	1-2	64
l_2	1-7	95
l_3	1-8	42
l_4	1-9	63
l_5	2-5	59
l_6	3-5	28
l_7	3-6	36
l_8	4-8	76
l_9	4-10	115
l_{10}	5-10	48
l_{11}	6-7	41
l_{12}	7-8	59
l_{13}	7-9	69
l_{14}	8-10	143

Supplementary Table 4: Annualized Cost of Prospective Transmission Lines

Line	2020	2021	2022	2023	2024	2025	2026	2027	2028	2029
l_1	128.08	126.80	125.54	124.28	123.04	121.81	120.59	119.38	118.19	117.01
l_2	189.49	187.60	185.72	183.86	182.03	180.21	178.40	176.62	174.85	173.11
l_3	83.89	83.05	82.22	81.40	80.58	79.78	78.98	78.19	77.41	76.63
l_4	127.15	125.88	124.62	123.37	122.14	120.92	119.71	118.51	117.33	116.15
l_5	117.46	116.28	115.12	113.97	112.83	111.70	110.58	109.48	108.38	107.30
l_6	56.09	55.53	54.98	54.43	53.88	53.34	52.81	52.28	51.76	51.24
l_7	72.70	71.97	71.25	70.54	69.84	69.14	68.45	67.76	67.08	66.41
l_8	151.90	150.38	148.87	147.38	145.91	144.45	143.01	141.58	140.16	138.76
l_9	230.91	228.60	226.31	224.05	221.81	219.59	217.40	215.22	213.07	210.94
l_{10}	95.35	94.39	93.45	92.51	91.59	90.67	89.77	88.87	87.98	87.10
l_{11}	82.26	81.44	80.63	79.82	79.02	78.23	77.45	76.67	75.91	75.15
l_{12}	118.44	117.25	116.08	114.92	113.77	112.63	111.51	110.39	109.29	108.20
l_{13}	139.27	137.88	136.50	135.13	133.78	132.44	131.12	129.81	128.51	127.23
l_{14}	288.37	285.48	282.63	279.80	277.00	274.23	271.49	268.78	266.09	263.43

Supplementary Table 5: Wind Turbine Parameters[7]

Parameter	Description	Value	Units
$P^{W,rated}$	Rated Power	4.2	MW
$u^{W,in}$	Cut-in wind speed	3	m/s
$u^{W,rated}$	Rated wind speed	12.5	m/s
$u^{W,out}$	Cut out wind speed	22.5	m/s
z	Rotor Diameter	150	m

Supplementary Table 6: Photovoltaic Cell Parameters

Parameter	Description	Value	Units
PMP^{ref}	Rated Maximum Power	0.25	kW
κ^{PV}	Power Temperature coefficient	3	%/°C
$NOCT$	Nominal operating cell temperature	41.5	°C
T^{ref}	Reference temperature	25	°C

Supplementary Table 7: Expected Value of Information and Value of Stochastic Solution. Shown are results for the Base, Kingdom of Saudi Arabia (KSA) Grid, Wind Only, and Photovoltaics (PV) Only cases.

	SP [M$]	WS [M$]	EVRS [M$]	EVPI [M$]	VSS [M$]
Base	4,608	4,185	4,724	423	115
KSA Grid	4,204	4,009	4,289	195	85
Wind Only	7,397	6,202	7,509	1,195	111
PV Only	16,803	13,412	16,864	3,391	60

SP: stochastic problem; WS: wait and see; EVRS: expected value of the reference scenario; EVPI: expected value of perfect information; VSS: value of the stochastic solution

Supplementary Table 8: Power sector generating capacity investments for 20% power consumption from desalination. Shown are results for the Base, Kingdom of Saudi Arabia (KSA) Grid, Wind Only, and Photovoltaics (PV) Only cases. Co-optimization reduces the total necessary power generating capacity in all cases.

Power Sector	Generation [GW]					
	CCGT	CSP	PV	Wind	Total	% Change of Total
	Independent/Co-Optimization					
Base	0 / 0	4.2 / 3.9	0 / 0	1.2 / 1.4	5.4 / 5.2	-3.0
KSA Grid	0 / 0	2.1 / 2.1	0 / 0	2.33 / 2.3	4.4 / 4.4	-1.0
Wind Only	0 / 0	0 / 0	0 / 0	12.2 / 12.1	12.2 / 12.1	-1.4
PV Only	0 / 0	0 / 0	42.4 / 40.4	0 / 0	42.4 / 40.4	-4.7

CCGT: Combined-cycle gas turbine; CSP: Concentrated solar power

Supplementary Table 9: Power sector energy storage capacity investments for 20% power consumption from desalination. Shown are results for the Base, Kingdom of Saudi Arabia (KSA) Grid, Wind Only, and Photovoltaics (PV) Only cases. Co-optimization enables a reduction in total power storage capacity needed to meet power demand for all cases.

Power Sector	Storage [GWh]				
	Batteries	HPS	TES	Total	% Change of Total
	Independent/Co-Optimization				
Base	0 / 0	0 / 0	49.9 / 46.5	49.9 / 46.5	-6.9
KSA Grid	0 / 0	0 / 0	25.4 / 25.6	25.4 / 25.6	-0.7
Wind Only	70.9 / 58.5	0 / 0	0 / 0	70.9 / 58.5	-17.5
PV Only	153.0 / 140.0	0 / 0	0 / 0	153.0 / 140.0	-8.5

HPS: Hydroelectric pump storage; TES: Thermal energy storage

Supplementary Table 10: Water sector capacity investments for 20% power consumption from desalination. Shown are results for the Base, Kingdom of Saudi Arabia (KSA) Grid, Wind Only, and Photovoltaics (PV) Only cases. Investment capacities for reverse osmosis (RO) and water tanks increase to supply for all cases when co-optimizing the power and water sectors.

Water Sector	Production [1000 m^3/h]			Storage [1000 m^3]		
	RO	Total	% Change of Total	Water Tanks	Total	% Change of Total
	Independent/Co-Optimization			Independent/Co-Optimization		
Base	147.2 / 148.3	147.2 / 148.3	0.7	3367. 4 / 3,821.0	3367. 4 / 3,821.0	13.5
KSA Grid	147.2 / 154.6	147.2 / 154.6	5.0	3367. 4 / 3,492.5	3367. 4 / 3,492.5	3.7
Wind Only	147.2 /158.9	147.2 /158.9	7.9	3367. 4 / 3,821.0	3367. 4 / 3,821.0	13.5
PV Only	147.2 / 207.5	147.2 / 207.5	40.9	3367. 4 / 3,496.4	3367. 4 / 3,496.4	3.8

Supplementary Table 11: Power sector generating capacity investments for 4% power consumption from desalination. Shown are results for the Base, Kingdom of Saudi Arabia (KSA) Grid, Wind Only, and Photovoltaics (PV) Only cases. In the Wind Only and Photovoltaics Only cases, co-optimization reduces the total necessary power generating capacity for cases without dispatchable generators.

Power Sector	Generation [GW]					
	CCGT	CSP	PV	Wind	Total	% Change of Total
	Independent/Co-Optimization					
Base	0 / 0	4.2 / 4.1	0 / 0	1.2 / 1.3	5.40 / 5.40	0
KSA Grid	0 / 0	2.1 / 2.0	0 / 0	2.3 / 2.4	4.4 / 4.4	0
Wind Only	0 / 0	0 / 0	0 / 0	12.3 / 12.4	12.3 / 12.4	-0.8
PV Only	0 / 0	0 / 0	42.4 / 41.8	0 / 0	42.4 / 41.8	-1.4

CCGT: Combined-cycle gas turbine; CSP: Concentrated solar power

Supplementary Table 12: Power sector energy storage capacity investments for 4% power consumption from desalination. Shown are results for the Base, Kingdom of Saudi Arabia (KSA) Grid, Wind Only, and Photovoltaics (PV) Only cases. Co-optimization enables a reduction in total storage capacity needed to meet power demand for all cases.

Power Sector	Storage [GWh]				
	Batteries	HPS	TES	Total	% Change of Total
	Independent/Co-Optimization				
Base	0 / 0	0 / 0	49.9 / 48.9	0	-2.0
KSA Grid	0 / 0	0 / 0	25.4 / 24.4	25.4 / 24.4	-3.9
Wind Only	70.9 / 68.2	0 / 0	0 / 0	70.9 / 68.2	-3.8
PV Only	153.0 / 149.8	0 / 0	0 / 0	153.0 / 149.8	-2.1

HPS: Hydroelectric pump storage; TES: Thermal energy storage

Supplementary Table 13: Water sector capacity investments for 4% power consumption from desalination. Shown are results for the Base, Kingdom of Saudi Arabia (KSA) Grid, Wind Only, and Photovoltaics (PV) Only cases. Investment capacities for reverse osmosis (RO) and water tanks increase to supply for all cases when co-optimizing the power and water sectors. An exception is RO for the Base case which exhibits a small decrease.

Water Sector	Production [1000 m^3/h]			Storage [1000 m^3]		
	RO	Total	% Change of Total	Water Tanks	Total	% Change of Total
	Independent/Co-Optimization			Independent/Co-Optimization		
Base	39.5 / 39.0	39.5 / 39.0	-1.3	894.5 / 932.4	894.5 / 932.4	4.2
KSA Grid	39.5 / 41.0	39.5 / 41.0	3.8	894.5 / 931.1	894.5 / 931.1	4.1
Wind Only	39.5 / 44.0	39.5 / 44.0	11.4	894.5 / 1,018.7	894.5 / 1,018.7	13.9
PV Only	39.5 / 44.5	39.5 / 44.5	12.7	894.5 / 931.1	894.5 / 931.1	4.1

Supplementary Table 14: Power sector cost measures for all cases when desalination accounts for 4% of the total power consumption. Shown are results for the Base, Kingdom of Saudi Arabia (KSA) Grid, Wind Only, and Photovoltaics (PV) Only cases. For the power sector, all systems experience a decrease in total cost due to decreased investment spending. However, systems lacking dispatchable power generators see the biggest savings.

Power Sector	Investment cost [M$]	Operating cost [M$]	Total cost [M$]	% Change of Total Cost
	Independent/Co-Optimization			
Base	1,705/1,688	299/283	2,004/1,971	-1.6
KSA Grid	1,146/1,132	359/ 360	1,505/1,492	-0.9
Wind Only	5,088/4,939	149/147	5,236/5,086	-2.9
PV Only	13,766/13,539	1,094/1,090	14,860/14,629	-1.6

Supplementary Table 15: Water sector cost measures for all cases when desalination accounts for 4% of the total power consumption. Shown are results for the Base, Kingdom of Saudi Arabia (KSA) Grid, Wind Only, and Photovoltaics (PV) Only cases. For the water sector, an increase in total costs resulting from higher investment spending is particularly evident in the PV Only and Wind Only cases.

Water Sector	Investment cost [M$]	Operating cost [M$]	Total cost [M$]	% Change of Total Cost
	Independent/Co-Optimization			
Base	97/96	633/634	730/730	0.0
KSA Grid	97/100	633/631	730/731	0.1
Wind Only	97/108	633/628	730/736	0.8
PV Only	97/108	633/630	730/738	1.1

Supplementary Table 16: System cost measures for all cases when desalination accounts for 4% of the total power consumption. Shown are results for the Base, Kingdom of Saudi Arabia (KSA) Grid, Wind Only, and Photovoltaics (PV) Only cases. System costs decrease for all cases when co-optimizing the power and water sectors.

System	Investment cost [M$]	Operating cost [M$]	Total cost [M$]	% Change of Total Cost
	Independent/Co-Optimization			
Base	1,802 / 1,784	932 / 917	2,734 / 2,701	-1.2
KSA Grid	1,243 / 1,232	992 / 991	2,235 / 2,223	-0.5
Wind Only	5,185 / 5,047	782 / 775	5,966 / 5,822	-2.4
PV Only	13,863 / 13,647	1,727 / 1,720	15,590 / 15,367	-1.4

Supplementary Table 17: Levelized cost of electricity (LCOE) and levelized cost of water (LCOW) for the Base, Kingdom of Saudi Arabia (KSA) Grid, Wind Only, and Photovoltaics (PV) only cases. Desalination accounts for 4% of the total power consumption.

	LCOE [$/MWh]		LCOW [$/m^3]	
	Independent/Co-Optimization	% Change	Independent/Co-Optimization	% Change
Base	17.95/17.86	-1	0.61/0.61	0
KSA Grid	13.34/13.25	-1	0.61/0.61	0
Wind Only	134.07/132.85	-1	0.61/0.62	2
PV Only	47.90/47.11	-2	0.61/0.62	2

Supplementary Table 18: Computation performance for two stochastic programming problems with a time horizon of ten years using: 1) representative periods; and 2) full-time resolution. Using representative periods, the model demonstrates greater computational efficiency than solving a full resolution model.

	Representative periods	Full-time resolution
Computation time [h]	6.54	336
Optimality gap [%]	0	43.2

Supplementary Table 19: Results for two stochastic programming problems with a time horizon of one year using: 1) representative periods; and 2) full time resolution.

	Representative periods	Full time resolution	Increment (%)
Investment capacity			
CSP [GW]	3.91	4.11	5.12
PV [GW]	0	0	0
Wind [GW]	1.36	1.39	2.21
CCGT [GW]	0	0	0
Battery [GWh]	0	0	0
HPS [GWh]	0	0	0
TES [GWh]	46.95	49.39	5.20
Computational results			
Computation time [h]	0.31	0.91	193.5
Optimality gap [%]	0	0	0

CSP: Concentrated solar power; PV: Photovoltaics; CCGT: Combined cycle gas turbine; HPS: Hydroelectric pump storage; TES: Thermal energy storage

Supplementary Table 20: Results for two deterministic problems with a time horizon of one year using: 1) representative periods; and 2) full time resolution.

	Representative periods	Full time resolution	Increment (%)
Investment capacity			
CSP [GW]	3.00	3.09	3.00
PV [GW]	0	0	0
Wind [GW]	1.22	1.24	1.64
CCGT [GW]	0	0	0
Battery [GWh]	0	0	0
HPS [GWh]	0	0	0
TES [GWh]	35.95	36.99	2.89
Computational results			
Computation time [h]	0.06	0.17	167
Optimality gap [%]	0	0	0

CSP: Concentrated solar power; PV: Photovoltaics; CCGT: combined cycle gas turbine; HPS: Hydroelectric pump storage; TES: Thermal energy storage

Supplementary Notes.

Supplementary note 1: Annualized Cost Calculations

Annualized cost is a metric that allows decision-makers to compare the cost-effectiveness of technologies with different lifespans. The annualized investment cost (AIC) of technology i, in year f, is a function of that year's overnight capital cost, $OCC_{i,f}$, and the technology's capital recovery factor, CRF_i:

$$AIC_{i,f} = OCC_{i,f} CRF_i. \tag{1}$$

The capital recovery factor is the net present value of payments over a fixed period – in this case, the lifespan, LTL_i, of the technology. It is calculated using,

$$CRF_i = \frac{r(1+r)^{LTL_i}}{(1+r)^{LTL^i}-1} \tag{2}$$

where r is the nominal interest rate.

Overnight investment and operating costs power generating technologies excluding CSP were obtained from the U.S. Energy Information Administration website.[1] For CSP and HPS, cost values were obtained from the National Renewable Energy Laboratory.[2] Overnight investment and operating costs power for desalination was obtained from Caldera et al. 2017.[3]

We present the annualized investment costs of the power generating and energy storage technologies in Supplementary Table 1. Supplementary Table 2 includes the annualized investment costs of the water systems technologies. Supplementary Table 4 includes the annualized investment cost of the prospective transmission lines.

Supplementary note 2: k-means Clustering Framework

The goal of k-means clustering is to select k number of representative periods/regions that minimizes the sum of squared distance between the rows, $\mathbf{p}_i$, of matrix $\widehat{\mathbf{P}}$ and their cluster centroid$\mathbf{c}_k$:

$$\phi^{pot} = \min_{\mathcal{C}_1 \dots \mathcal{C}_k} \sum_{k \in \mathcal{K}} \sum_{i \in \mathcal{C}} \left|\left|\boldsymbol{p}_i - \boldsymbol{c}_k\right|\right|^2, \tag{3}$$

where $\mathcal{C}$ is a set of k clusters and $\mathcal{K}$ is a set of k centroids. The k-means approach most commonly used is Lloyd's algorithm[4], which begins by selecting k arbitrary centroids, chosen uniformly at random from the normalized dataset, $\widehat{\mathbf{P}}$. Each row is assigned to the nearest centroid, determined by calculating the Euclidean distance between a given row to the k possible centroids. This step forms the initial clusters. The centroid is then recalculated for each cluster, taking the mean of all data points in the cluster.

The assignment and recalculation steps are repeated until the total distance, ϕ^{pot}, converges. However, despite its simplicity, the disadvantage of Lloyd's algorithm is that it is not always accurate and may generate inappropriate clusters.[5] The initialization of the centroids has a high impact on finding optimal clusters and centroids.

Arthur & Vassilvitskii[6] proposed an alternative, k-means++, that involved initializing Lloyd's k-means algorithm with random starting centers with specific probabilities proportional to their contribution to the overall potential. The first centroid, $\mathbf{c}_1$, is selected uniformly from the dataset$\widehat{\mathbf{P}}$. The next center, $\mathbf{c}_2$, is chosen at random from, $\widehat{\mathbf{P}}$, where $\mathbf{c}_2 = \mathbf{p}' \in \widehat{\mathbf{P}}$ with a probability of $\frac{D(\mathbf{p}')^2}{\sum_{\mathbf{p} \in \widehat{\mathbf{P}}} D(\mathbf{p})^2}$. Here $D(\mathbf{p})^2$ denotes the shortest distance between row $\mathbf{p}$ to the closest center we have. This is repeated until all k centroids are selected. The remainder of Lloyd's k-means algorithm (reassignment and recalculation) is subsequently performed.

Supplementary note 3: Selection of representative regions

Spatial clustering is used to delineate regions with similar climate conditions: wind speed, DNI, and temperature. The dataset comprises 4270 (number of location) time series, each 11.5 years long for each of the 3threeattributes. The spatial resolution is 5 km. The original data set was a 3-dimensional, 4270 locations × 100,656 hours × 3 attributes matrix. It was reshaped as:

$$\boldsymbol{P}^{spat} = [\boldsymbol{P}_{ws} \quad \boldsymbol{P}_{d} \quad \boldsymbol{P}_{t}] \, , \tag{4}$$

a 2-dimensional 4270 locations × M (302184), where m is composed of 100,656 hours for each of the 3 attributes.

For any given location, the time series corresponds to the values of the attributes throughout the 100,656 hours. This ensures that clustered locations have similar patterns for all attributes. Each attribute group is normalized using the z-scoring full scope normalization to produce a normalized matrix,

$$\widehat{\boldsymbol{P}}^{spat} = \left[\frac{1}{\sigma_{ws}}(\boldsymbol{P}_{ws} - \mu_{ws}) \quad \frac{1}{\sigma_{d}}(\boldsymbol{P}_{d} - \mu_{d}) \quad \frac{1}{\sigma_{t}}(\boldsymbol{P}_{t} - \mu_{t})\right]. \tag{5}$$

To ensure optimal clustering and reduce the value of the potential equation, the k-means++ method, for a given k, is initialized with 1000 random starting points. The clusters of the replicate with the best sum of the distances are selected as the optimal clusters. Given the nature of the dataset, clusters may not be continuous spatially, as seen in Supplementary Figure 1a. Points that are discontinuous or "wandering" within other clusters are manually reclassified. The distance between cluster centroid is calculated with,

$$C_{i,j}^{dist} = \left|\left|\boldsymbol{c}_i - \boldsymbol{c}_j\right|\right|^2, \tag{6}$$

to determine which regions are most similar.

Wandering points surrounded entirely by a cluster get reassigned to that cluster. A wandering point between two or more clusters is reassigned to the cluster with the shortest distance to its original assignment. The centroids of all clusters are subsequently recalculated. Supplementary Figure 1b shows the cluster distribution after reassignment takes place.

For spatial clustering, the cluster centroids are chosen as the time series for the representative regions. The centroids are merged into a, 10 locations × 302184 (100,656 hours × 3 attributes) matrix and reshaped into a three dimensional, 10 locations × 100,656 hours × 3 attributes matrix that is used for temporal clustering.

Supplementary note 4: Selection of representative days

For representative periods, we consider the attributes from the spatial clustering (wind speed, DNI, and temperature) and water demand and power load. In GEP models, we would ideally know the power and water demand of the original locations and subsequently include these attributes as part of the spatial clustering procedure. However, when only total demands are known, they are excluded from spatial clustering.

Once the representative regions are obtained, they are assigned power and water demands based on knowledge of what will/does exist in those regions (e.g., residences, industry, mixed, greenfield). The matrix from the spatial cluster is merged with a, 10 locations × 100,656 hours × 2, matrix containing the demand and water profiles for the regions. This 3-dimensional, 100,656 hours × 10 locations × 5 attributes matrix is reshaped as:

$$\widehat{\boldsymbol{P}}^{temp} = [\boldsymbol{P}_{ws} \quad \boldsymbol{P}_{d} \quad \boldsymbol{P}_{t} \quad \boldsymbol{P}_{wt} \quad \boldsymbol{P}_{e}], \qquad (7)$$

a 2-dimensional, 4194 days ×M (1200) matrix, where M contains the hours per day (24), locations (10), and attributes (5).

For a given day, the signal pattern corresponds to the value of the attributes throughout the day for all locations. This was done to ensure that the selected representative days best capture the spatial and temporal variability between all attributes together. Each attribute group is normalized using the z-scoring full scope normalization scope us to produce the normalized matrix,

$$\widehat{\boldsymbol{P}}^{temp} = \left[\frac{1}{\sigma_{ws}}(\boldsymbol{P}_{ws} - \mu_{ws}) \quad \frac{1}{\sigma_{d}}(\boldsymbol{P}_{d} - \mu_{d}) \quad \frac{1}{\sigma_{t}}(\boldsymbol{P}_{t} - \mu_{t}) \quad \frac{1}{\sigma_{wt}}(\boldsymbol{P}_{wt} - \mu_{wt}) \quad \frac{1}{\sigma_{e}}(\boldsymbol{P}_{e} - \mu_{e})\right]. \qquad (8)$$

To ensure optimal clustering and reduce the value of the potential equation, the *k*-means++ method for a given *k* is initialized with 1000 random starting points. The clusters of the replicate with the best sum of the distances are selected as the optimal clusters.

For visual simplicity, Supplementary Figure 2 shows the *k*-means clustering output of the temperature portion of the dataset. In this case, $k = 5$.

In the representation stage, representative days that minimize the Euclidean distance to its corresponding cluster centroid are selected. Weights of the representative days are calculated by dividing the number of days in their associated cluster by the number of days in the entire dataset.

Supplementary note 5: Renewable Power Capacity Factors

Wind Power

The electrical power generated by a wind turbine is a function of the power produced by the wind upon entering the turbine itself and a power coefficient at the given wind speed. We present the power outputs of the Vestas 150-4.2MW turbine. The wind turbine parameters are shown in Supplementary Table 5.

The power coefficients (Cp), provided by the turbine manufacturer at varying winds speeds, are a measure of efficiency represented as the product of turbine efficiency, the efficiency of the shaft bearings and gears, and the generator efficiency. We fit the given Cp values to an exponential curve:

$$Cp = \sum_{i=1}^{3} a_i e^{-\left(\frac{u-b_i}{c_i}\right)^2}, \quad (9)$$

where, a_i, b_i, c_i are the coefficient of the regression, and u is the wind speed.

The power entering the wind turbine,

$$p_t^{W,in} = \frac{\rho^{air} A^{Swept} u_t^3}{2} \quad , \quad (10)$$

is a function of air density, ρ^{air}, times the swept area of the turbine blade, A^{Swept}, and the wind speed, u, cubed. Air density generally varies according to pressure and temperature; however, we assume it to be constant at 1.225 kg/m^3. The area swept by the turbine is given by:

$$A^{Swept} = \frac{\pi z^2}{4} \quad . \quad (11)$$

The electrical power produced by the turbine,

$$p_t^{W,out} = Cp_t p_t^{W,in}, \quad (12)$$

is obtained by multiplying the power coefficients by the power entering the turbine.

To obtain the capacity factor of the wind turbine,

$$F_t^W = \frac{p_t^{W,out}}{p^{W,rated}}, \quad (13)$$

we divide power output by the rated power capacity.

PV Solar Power

The electrical power produced by a PV panel at a given hour, p_t^{PV}, is a function of temperature, T^{cell}, and solar irradiance, DNI_t , received by the cell[8]:

$$p_t^{PV} = PMP^{ref} \frac{DNI_t}{1000} [1 + \kappa^{PV} (T_t^{cell} - T^{ref})]. \quad (14)$$

The temperature of the cell depends on the ambient temperature, T_t^{amb}, and the solar irradiance:

$$T_t^{cell} = T_t^{amb} + \frac{NOCT-20}{800} DNI_t. \quad (15)$$

The photovoltaic capacity factor, F_t^{PV}, is calculated using the following equation,

$$F_t^{PV} = \frac{p_t^{PV}}{PMP^{ref}} . \quad (16)$$

The solar panel parameters are shown in Supplementary Table 6.

Supplementary note 6: Mathematical Formulation of the Stochastic Programming Model
Nomenclature
Indices

b	Battery farms
c	CSP farms
f	Years
h	HPS plants
j	CCGT plant
k	Water tank units
l	Transmission lines
$lf(f)$	Last year
$lo(o)$	Last day
$lt(t)$	Last hour
n	Nodes
o	Representative days
$rl(l)$	Receiving-end node of transmission line l
s	PV solar farms
$sl(l)$	Sending-end node of transmission line l
t	Hours
w	Wind farms
z	RO desalination plants
δ	Scenarios

Sets

Ω^B	Set of battery plants
Ω^B_n	Set of battery plants located at node n
Ω^C	Set of CSP farms
Ω^C_n	Set of CSP farms located at node n
Ω^H	Set of HPS plants
Ω^H_n	Set of HPS plants located at node n
Ω^J	Set of CCGT plants
Ω^J_n	Set of CCGT plants located at node n
Ω^K	Set of water tank units
Ω^K_n	Set of water tank units located at node n
Ω^L	Set of existing transmission lines
Ω^{L+}	Set of new transmission lines
Ω^P	Set of photovoltaic solar farms
Ω^P_n	Set of photovoltaic solar farms located at node n
Ω^W	Set of wind farms
Ω^W_n	Set of wind farms located at node n
Ω^Z	Set of RO desalination plants
Ω^Z_n	Set of RO desalination plants located at node n

Parameters

A^Z_z	Conversion factor between water produced and electricity consumed [MWh/m^3]
B_l	Susceptance of transmission line l [S]
C^B_b	Operating cost of battery plant b [\$/MWh]
C^H_h	Operating cost of HPS plant h [\$/MWh]

C_k^K	Operating cost of water tank k [\$/m^3]
C_j^J	Operating cost of CCGT j [\$/MWh]
C_c^{PB}	Operating cost of CSP farms c [\$/MWh]
C_p^{PV}	Operating cost of PV solar farms p [\$/MWh]
C_w^W	Operating cost of wind farm w [\$/MWh]
C_k^K	Operating cost of RO desalination plant z [\$/m^3]
$\bar{E}_{b,f}^{B,inv,max}$	Maximum capacity that can be built for CSP farm c in year f [MWh]
$F_l^{L,cap}$	Capacity of transmission line l [MW]
$F_{\delta,p,f,o,t}^{PV}$	Capacity factor of PV solar farm p for scenario δ, year f, day o and hour t [p.u.]
$F_{\delta,w,f,o,t}^{W}$	Capacity factor of wind farm w for scenario δ, year f, day o and hour t [p.u.]
$H_c^{TES,PB}$	Hours of TES energy storage [hrs]
H_h	Water head in plant HPS h [m]
$I_{b,f}^B$	Annualized investment cost of battery plant b in year f [\$/MWh]
$I_f^{GE,max}$	Annual investment budget for power sector [\$]
$I_f^{GW,max}$	Annual investment budget for water sector[\$]
$I_{h,f}^H$	Annualized investment cost of HPS plant h in year f [\$/MW]
$I_{j,f}^J$	Annualized investment cost of CCGT plant j in year f [\$/MW]
$I_{k,f}^K$	Annualized investment cost of water tank k in year f [\$/m^3]
$I_{l,f}^N$	Annualized investment cost of prospective transmission line l in year f [\$]
$I_{c,f}^{PB}$	Annualized investment cost of the power block of CSP farm c in year f [\$/MWh]
$I_{p,f}^{PV}$	Annualized investment cost of PV solar farm p in year f [\$/MWh]
$I_{w,f}^W$	Annualized investment cost of wind farm w in year f [\$/MWh]
$I_{z,f}^K$	Annualized investment cost of RO desalination plant z in year f [\$/MWh]
K_h^{pump}	Power consumption factor [MWh/m^4]
$K_h^{turbine}$	Power generation factor [MWh/m^4]
$M_c^{SF,PB}$	Solar multiple of CSP farm c [p.u.]
$P_{\delta,n,f,o,t}^D$	Electricity demand at node n, for scenario δ, year f, day o and hour t [MW]
$\bar{P}_{j,f}^{B,inv,max}$	Maximum capacity that can be built of CCGT plant j in year f [MW]
$\bar{P}_{C,f}^{PB,inv,max}$	Maximum power block (PB) capacity that can be built for CSP farm c in year c [MW]
$\bar{P}_{p,f}^{PV,inv,max}$	Maximum capacity that can be built of PV solar farm p in year f [MW]
$\bar{P}_{j,f}^{W,inv,max}$	Maximum capacity that can be built of wind farm w in year f [MW]
$Q_{\delta,n,f,o,t}^D$	Water demand at node n, for scenario δ, year f, day o and hour t [m^3 /h]
$\bar{Q}_{z,f}^{Z,inv,max}$	Maximum capacity that can be built of RO desal plant z in year f [m^3 /h]
R_j^D	Ramping-down limit of CCGT j [MW]
R_j^U	Ramping-up limit of CCGT j [MW]
$\bar{S}_{k,f}^{K,inv,max}$	Maximum capacity that can be built for tank k in year f [m^3]
$\bar{S}_f^{K,totcap}$	Maximum total capacity for all tanks in year f [m^3]
$\bar{V}_{h,f}^{H,inv,max}$	Maximum reservoir capacity that can be built for HPS plant h in year f [m^3]
$\underline{V}_{h,f}^{H,inv,min}$	Minimum reservoir capacity that can be built for HPS plant h in year f [m^3]

α_o	Weight of the representative day o. A given weight equals the number of days in a year that a representative day represents.
β_δ	Probability of scenario δ
γ_b	Minimum energy level coefficient of battery plant b [p.u.]
ϵ_b	Maximum charge/discharge coefficient of battery plant b [p.u.]
ζ_b^{chrg}	Charging efficiency coefficient of battery plant b [p.u.]
ζ_b^{dchrg}	Discharging efficiency coefficient of battery plant b [p.u.]
η_c	Minimum energy level coefficient of TES for CSP farm c [p.u.]
ι_c	Power block efficiency coefficient of CSP farm c [p.u.]
μ_h	Minimum water level coefficient of reservoir h [p.u.]
ν_h	Maximum pump/turbine coefficient of HPS plant h [p.u.]
ξ_b^{pump}	Pumping efficiency coefficient of HPS plant b [p.u.]
$\xi_b^{turbine}$	Turbining efficiency coefficient of HPS plant b [p.u.]
τ_j	Minimum power output coefficient of CCGT plant j [p.u.]
ρ_k	Minimum stored water level coefficient of water tank k [p.u.]
σ_k	Maximum inflow/outflow coefficient of water tank unit k [p.u.]
ϕ_z	Minimum water output coefficient of desalination plant z [p.u.]
ψ_z	Total renewable capacity coefficient [p.u.]

Binary Variables

$a_{l,f}$	Equal to 1 after transmission line l is built in year f, and 0 otherwise
$b_{l,f}$	Equal to 1 if transmission line l is built in year f, and 0 otherwise
$a_{\delta,j,f,o,t}$	Equal to 1 if CCGT unit j is online for scenario δ, year f, day o, and hour t, and 0 otherwise

Deterministic Continuous Variables

$E_{B,f}^{B,cap}$	Cumulative built capacity of battery unit b in year f [MWh]
$E_{B,f}^{B,inv}$	Capacity battery unit b built in year f [MWh]
$P_{j,f}^{J,cap}$	Cumulative built capacity of CCGT unit j in year f [MW]
$P_{j,f}^{J,inv}$	Capacity CCGT unit j built in year f [MW]
$P_{c,f}^{PB,cap}$	Cumulative built of PB for CSP farm j in year f [MW]
$P_{c,f}^{PB,inv}$	Capacity of PB for CSP farm c built in year f [MW]
$P_{p,f}^{PV,cap}$	Cumulative built capacity PV farm p in year f [MW]
$P_{p,f}^{PV,inv}$	Capacity of PV farm p built in year f [MW]
$P_{w,f}^{W,cap}$	Cumulative built capacity of wind farm w in year f [MW]
$P_{w,f}^{W,inv}$	Capacity of wind farm p built in year f [MW]
$Q_{z,f}^{Z,cap}$	Cumulative built capacity of RO desalination plant z in year f [m^3]
$Q_{z,f}^{Z,inv}$	Capacity of RO desalination z built in year f [m^3]
$S_{k,f}^{K,cap}$	Cumulative built capacity of water tank k in year f [m^3]
$S_{k,f}^{K,inv}$	Capacity of water tank k built in year f [m^3]
$V_{h,f}^{H,cap}$	Cumulative reservoir capacity of HPS unit h in year f [m^3]
$V_{h,f}^{H,inv}$	Capacity of HPS unit h in year f [m^3]

Stochastic Continuous Variables

$E^{B}_{\delta,b,f,o,t}$	Energy level of battery b in scenario δ, year f, day o, and hour t [MWh]
$E^{TES}_{\delta,b,f,o,t}$	Energy level of TES for CSP farm c in scenario δ, year f, day o, and hour t [MWh]
$F^{L}_{\delta,l,f,o,t}$	Power flow through transmission line l in scenario δ, year f, day o, and hour t [MW]
$p^{B,chrg}_{\delta,b,f,o,t}$	Charging power from battery unit b in scenario δ, year f, day o, and hour t [MW]
$p^{B,dchrg}_{\delta,b,f,o,t}$	Discharging power from battery unit b in scenario δ, year f, day o, and hour t [MW]
$p^{CSP,spill}_{\delta,c,f,o,t}$	Power spilled by CSP solar farm c in scenario δ, year f, day o, and hour t [MW]
$p^{H,pump}_{\delta,h,f,o,t}$	Power consumed by HPS unit h in scenario δ, year f, day o, and hour t [MW]
$p^{H,turbine}_{\delta,h,f,o,t}$	Power produced by HPS unit h in scenario δ, year f, day o, and hour t [MW]
$p^{J}_{\delta,j,f,o,t}$	Power produced by CCGT unit in scenario δ, year f, day o, and hour t [MW]
$p^{PB}_{\delta,c,f,o,t}$	Power produced by PB of CSP plant c in scenario δ, year f, day o, and hour t [MW]
$p^{PV,spill}_{\delta,p,f,o,t}$	Power spilled by PV solar farm p in scenario δ, year f, day o, and hour t [MW]
$p^{SF}_{\delta,c,f,o,t}$	Power produced by SF of CSP plant c for scenario δ, year f, day o, and hour t [MW]
$p^{TES,inj}_{\delta,c,f,o,t}$	Power stored in TES of CSP plant c for scenario δ, year f, day o, and hour t [MW]
$p^{TES,out}_{\delta,c,f,o,t}$	Power withdrawn from TES of CSP plant c for scenario δ, year f, day o, and hour t [MW]
$p^{wind,spill}_{\delta,w,f,o,t}$	Power spilled by wind farm w for scenario δ, year f, day o, and hour t [MW]
$q^{H,pump}_{\delta,h,f,o,t}$	Pumped flow of water by HPS unit h for scenario δ, year f, day o, and hour t [MW]
$q^{H,turbine}_{\delta,h,f,o,t}$	Turbined flow of water by HPS unit h for scenario δ, year f, day o, and hour t [m^3/h]
$q^{K,in}_{\delta,k,f,o,t}$	Inflow of water from water tank k for scenario δ, year f, day o, and hour t [m^3/h]
$q^{K,out}_{\delta,k,f,o,t}$	Outflow of water from water tank k for scenario δ, year f, day o, and hour t [m^3/h]
$q^{Z}_{\delta,z,f,o,t}$	Water desalinated by plant h for scenario δ, year f, day o, and hour t [m^3/h]
$S^{K}_{\delta,k,f,o,t}$	Water level of tank unit k for scenario δ, year f, day o, and hour t [m^3]
$V^{H}_{\delta,h,f,o,t}$	Reservoir level of unit h for scenario δ, year f, day o, and hour t [m^3]
$w_{\delta,z,f,o,t}$	Product of binary variable ($u_{\delta,z,f,o,t}$) and continuous variable () [MW]
$\theta_{\delta,n,f,o,t}$	Voltage angle at node n for scenario δ, year f, day o, and hour t [rad]

Objective Function

$$\min_{\Delta} \sum_f (capex_f^{power} + capex_f^{water}) + \sum_\delta \beta_\delta \sum_f \sum_o \alpha_o \sum_{t=1}^{24} (opex_{\delta,f,o,t}^{water} + opex_{\delta,f,o,t}^{water}) \quad (17)$$

where

$$capex_f^{power} = \sum_{l\in\Omega^{L+}} I_{lf}^N b_{lf} + \sum_{b\in\Omega^B} I_{b,f}^B E_{b,f}^{B,inv} + \sum_{c\in\Omega^C} I_{c,f}^{PB} P_{c,f}^{PB,inv} + \sum_{j\in\Omega^J} I_{j,f}^J P_{j,f}^{J,inv} + \sum_{h\in\Omega^H} I_{hf}^H \left(K_h^{turbine} v_h V_{h,f}^{H,inv} H_h\right) + + \sum_{p\in\Omega^P} I_{p,f}^{PV} P_{p,f}^{PV,inv} + \sum_{w\in\Omega^W} I_{w,f}^W P_{w,f}^{W,inv} \quad (18)$$

$$capex_f^{water} = \sum_{k\in\Omega^K} I_{k,f}^K S_{k,f}^{K,inv} + \sum_{z\in\Omega^Z} I_{z,f}^Z Q_{z,f}^{Z,inv} \quad (19)$$

$$opex_{\delta,f,o,t}^{power} = \sum_{b\in\Omega^B} C_b^B \left(E_{\delta,b,f,o,t}^{B,charge} + E_{\delta,b,f,o,t}^{B,discharge}\right) + \sum_{c\in\Omega^C} C_c^{PB} p_{\delta,c,f,o,t}^{PB} + \sum_{h\in\Omega^H} C_h^H \left(p_{\delta,h,f,o,t}^{H,pump} + p_{\delta,h,f,o,t}^{turbine}\right) + \sum_{j\in\Omega^J} C_{\delta,j,f,o,t}^J p_{j,f}^{J,inv} + \sum_{p\in\Omega^P} C_p^{PV} F_{\delta,p,f,o,t}^{PV} P_{\delta,p,f,o,t}^{PV,cap} + \sum_{w\in\Omega^W} C_w^W F_{\delta,w,f,o,t}^W P_{\delta,w,f,o,t}^{W,cap} \quad (20)$$

$$opex_{\delta,f,o,t}^{water} = \sum_{k\in\Omega^K} C_k^K \left(q_{\delta,k,f,o,t}^{K,in} + q_{\delta,k,f,o,t}^{K,out}\right) + \sum_{z\in\Omega^Z} I_{z,f}^Z Q_{z,f}^{Z,inv} \quad (21)$$

$$\Delta = \left\{ \begin{array}{c} a_{lf}, b_{lf}, u_{\delta,j,f,o,t}, E_{b,f}^{B,cap}, E_{b,f}^{B,inv}, E_{c,f}^{TES,cap}, P_{j,f}^{J,cap}, P_{j,f}^{J,inv}, P_{c,f}^{PB,cap}, P_{c,f}^{PB,inv}, \\ P_{p,f}^{PV,cap}, P_{p,f}^{PV,inv}, P_{w,f}^{W,cap}, P_{w,f}^{W,inv}, Q_{z,f}^{Z,cap}, Q_{z,f}^{Z,inv}, S_{k,f}^{K,cap}, S_{k,f}^{K,inv}, V_{h,f}^{H,cap}, V_{h,f}^{H,inv}, \\ E_{\delta,b,f,o,t}^B, E_{\delta,b,f,o,t}^{TES}, F_{\delta,l,f,o,t}^L, p_{\delta,b,f,o,t}^{B,chrg}, p_{\delta,b,f,o,t}^{B,dchrg}, p_{\delta,c,f,o,t}^{CSP,spill}, p_{\delta,h,f,o,t}^{H,pump}, p_{\delta,h,f,o,t}^{H,turbine}, \\ p_{\delta,j,f,o,t}^J, p_{\delta,c,f,o,t}^{PB}, p_{\delta,p,f,o,t}^{PV,spill}, p_{\delta,c,f,o,t}^{SF}, p_{\delta,c,f,o,t}^{TES,inj}, p_{\delta,c,f,o,t}^{TES,out}, p_{\delta,w,f,o,t}^{wind,spill}, q_{\delta,h,f,o,t}^{H,pump} \\ q_{\delta,h,f,o,t}^{H,turbine}, q_{\delta,k,f,o,t}^{K,in}, q_{\delta,k,f,o,t}^{K,out}, q_{\delta,z,f,o,t}^Z, S_{\delta,k,f,o,t}^K, V_{\delta,h,f,o,t}^H, w_{\delta,z,f,o,t}, \theta_{\delta,n,f,o,t} \end{array} \right\} \quad (22)$$

Annual Power Generating Capacities Built

$$0 \le P_{c,f}^{PB,inv} \le \bar{P}_{c,f}^{PB,inv,max} \qquad c \in \Omega^C, \forall n, \forall f \quad (23)$$

$$0 \le P_{j,f}^{J,inv} \le \bar{P}_{j,f}^{J,inv,max} \qquad j \in \Omega^J, \forall n, \forall f \quad (24)$$

$$0 \le P_{p,f}^{PV,inv} \le \bar{P}_{p,f}^{PV,inv,max} \qquad p \in \Omega^P, \forall n, \forall f \quad (25)$$

$$0 \le P_{w,f}^{W,inv} \le \bar{P}_{w,f}^{W,inv,max} \qquad w \in \Omega^W, \forall n, \forall f \quad (26)$$

$$0 \le E_{b,f}^{B,inv} \le \bar{E}_{b,f}^{B,inv,max} \qquad b \in \Omega^B, \forall n, \forall f \quad (27)$$

$$y_{h,f} \bar{V}_{h,f}^{H,inv,min} \le V_{h,f}^{H,inv} \le y_{h,f} \bar{V}_{hf}^{H,inv,max} \qquad h \in \Omega^H, \forall n, \forall f \quad (28)$$

$$\sum_f y_{h,f} = 1 \qquad h \in \Omega^H, \forall n \quad (29)$$

Annual Transmission Lines Built

$$a_{l,f} \ge a_{l,f-1} \qquad l \in \Omega^{L+}, \forall f \quad (30)$$

$$b_{l,f} = a_{l,f} \qquad l \in \Omega^{L+}, f = 1 \quad (31)$$

$$b_{l,f} = a_{l,f} - a_{l,f} \qquad l \in \Omega^{L+}, f > 1 \quad (32)$$

Annual Water Producing Capacities Built

$$0 \leq Q_{z,f}^{Z,inv} \leq \bar{Q}_{z,f}^{Z,inv,max} \qquad z \in \Omega^{z}, \forall n, \forall f \qquad (33)$$

$$0 \leq S_{k,f}^{K,inv} \leq \bar{S}_{k,f}^{K,inv,max} \qquad k \in \Omega^{K}, \forall n, \forall f \qquad (34)$$

Capacity Balancing Between Years

$$P_{c,f}^{PB,cap} = P_{c,f-1}^{PB,cap} + P_{c,f}^{PB,inv} \qquad c \in \Omega_n^{C}, \forall n, f = 1 \qquad (35)$$

$$P_{c,f}^{PB,cap} = P_{c,f-1}^{PB,cap} + P_{c,f}^{PB,inv} \qquad c \in \Omega_n^{C}, \forall n, f > 1 \qquad (36)$$

$$P_{j,f}^{J,cap} = P_{j,f}^{J,inv} \qquad j \in \Omega_n^{J}, \forall n, f = 1 \qquad (37)$$

$$P_{j,f}^{J,cap} = P_{j,f-1}^{J,cap} + P_{j,f}^{J,inv} \qquad j \in \Omega_n^{J}, \forall n, f > 1 \qquad (38)$$

$$PP_{p,f}^{PV,cap} = P_{p,f}^{PV,inv} \qquad p \in \Omega_n^{P}, \forall n, f = 1 \qquad (39)$$

$$P_{p,f}^{PV,cap} = P_{p,f-1}^{PV,cap} + P_{p,f}^{PV,inv} \qquad p \in \Omega_n^{P}, \forall n, f > 1 \qquad (40)$$

$$P_{w,f}^{W,cap} = P_{w,f}^{W,inv} \qquad w \in \Omega_n^{W}, \forall n, f = 1 \qquad (41)$$

$$P_{w,f}^{W,cap} = P_{w,f-1}^{W,cap} + P_{w,f}^{W,inv} \qquad w \in \Omega_n^{W}, \forall n, f > 1 \qquad (42)$$

$$E_{b,f}^{B,cap} = E_{b,f}^{B,inv} \qquad b \in \Omega_n^{B}, \forall n, f = 1 \qquad (43)$$

$$E_{b,f}^{B,cap} = E_{b,f-1}^{B,cap} + E_{b,f}^{B,inv} \qquad b \in \Omega_n^{B}, \forall n, f > 1 \qquad (44)$$

$$V_{h,f}^{H,cap} = V_{hf}^{H,inv} \qquad h \in \Omega_n^{H}, \forall n, f = 1 \qquad (45)$$

$$V_{h,f}^{H,cap} = V_{hf-1}^{H,cap} + V_{h,f}^{H,inv} \qquad h \in \Omega_n^{H}, \forall n, f > 1 \qquad (46)$$

$$Q_{z,f}^{Z,cap} = Q_{z,f}^{Z,inv} \qquad z \in \Omega_n^{Z}, \forall n, f = 1 \qquad (47)$$

$$Q_{z,f}^{Z,cap} = Q_{z,f-1}^{Z,cap} + Q_{z,f}^{Z,inv} \qquad z \in \Omega_n^{Z}, \forall n, f > 1 \qquad (48)$$

$$S_{k,f}^{K,cap} = S_{k,f}^{K,inv} \qquad k \in \Omega_n^{K}, \forall n, f = 1 \qquad (49)$$

$$S_{k,f}^{K,cap} = S_{k,f-1}^{K,cap} + S_{k,f}^{K,inv} \qquad k \in \Omega_n^{K}, \forall n, f > 1 \qquad (50)$$

$$\sum_{k} S_{k,f}^{K,cap} \leq S_{f}^{K,totalcap} \qquad \forall f \qquad (51)$$

Investment Budget of Power Sector

$$\sum_{c\in\Omega^C} I_{c,f}^{PB} P_{c,f}^{PB,inv} + \sum_{h\in\Omega^H} I_{h,f}^{H} \left(K_h^{turbine} v_h V_{h,f}^{H,inv} H_h\right) + \sum_{j\in\Omega^J} I_{j,f}^{J} P_{j,f}^{J,inv} + \sum_{p\in\Omega^P} I_{p,f}^{PV} P_{p,f}^{PV,inv} + \sum_{w\in\Omega^W} I_{w,f}^{W} P_{w,f}^{W,inv} + \sum_{b\in\Omega^B} I_{b,f}^{B} P_{b,f}^{B,inv} \leq I_{f}^{GE,max} \qquad \forall f \qquad (52)$$

Investment Budget of Water Sector

$$\sum_{k\in\Omega^K} I_{k,f}^{K} S_{k,f}^{K,inv} + \sum_{z\in\Omega^Z} I_{z,f}^{Z} Q_{z,f}^{Z,inv} \leq I_f^{GW,max} \quad \forall f \quad (53)$$

CCGT Plant Operations

$$\tau_j P_{j,f}^{J,cap} u_{\delta,j,f,o,t} \leq p_{\delta,j,f,o,t}^{J} \leq P_{j,f}^{J,cap} u_{\delta,j,f,o,t} \quad \forall\delta, j\in\Omega_n^J, \forall n, \forall f, \forall o, \forall t \quad (54)$$

$$p_{\delta,j,f,o,t}^{J} \leq R_j^U \quad \forall\delta, j\in\Omega_n^J, \forall n, f=1, o=1, t=1 \quad (55)$$

$$p_{\delta,j,f,o,t}^{J} - p_{\delta,j,f-1,o=lo(o),t=lt(t)}^{J} \leq R_j^U \quad \forall\delta, j\in\Omega_n^J, \forall n, f>1, o=1, t=1 \quad (56)$$

$$p_{\delta,j,f,o,t}^{J} - p_{\delta,j,f,o-1,t=lt(t)}^{J} \leq R_j^U \quad \forall\delta, j\in\Omega_n^J, \forall n, \forall f, o>1, t=1 \quad (57)$$

$$p_{\delta,j,f,o,t}^{J} - p_{\delta,j,f,o,t-1}^{J} \leq R_j^U \quad \forall\delta, j\in\Omega_n^J, \forall n, \forall f, \forall o, t>1 \quad (58)$$

$$p_{\delta,j,f,o,t}^{J} \leq R_j^D \quad \forall\delta, j\in\Omega_n^J, \forall n, f=1, o=1, t=1 \quad (59)$$

$$p_{\delta,j,f-1,o=lo(o),t=lt(t)}^{J} - p_{\delta,j,f,o,t}^{J} \leq R_j^D \quad \forall\delta, j\in\Omega_n^J, \forall n, f>1, o=1, t=1 \quad (60)$$

$$p_{\delta,j,f,o-1,t=lt(t)}^{J} - p_{\delta,j,f,o,t}^{J} \leq R_j^D \quad \forall\delta, j\in\Omega_n^J, \forall n, \forall f, o>1, t=1 \quad (61)$$

$$p_{\delta,j,f,o,t-1}^{J} - p_{\delta,j,f,o,t}^{J} \leq R_j^D \quad \forall\delta, j\in\Omega_n^J, \forall n, \forall f, \forall o, t>1 \quad (62)$$

CSP Farm Operations

$$P_{c,f}^{SF,cap} = \frac{M_c^{SF,PB} P_{c,f}^{PB,cap}}{\iota_c} \quad \forall c, \forall n, \forall f \quad (63)$$

$$E_{c,f}^{TES,cap} = \frac{H_c^{TES,PB} P_{c,f}^{PB,cap}}{\iota_c} \quad \forall c, \forall n, \forall f \quad (64)$$

$$p_{\delta,c,fo,t}^{SF} = \frac{DNI_{\delta,c,f,o,t} P_{c,f}^{SF,cap}}{1000\ W/m^2} \quad \forall\delta, c\in\Omega_n^C, \forall n, \forall f, \forall o, \forall t \quad (65)$$

$$E_{\delta,c,f,o,t}^{TES} = p_{\delta,c,f,o,t}^{TES,inj} - p_{\delta,c,f,o,t}^{TES,out} \quad \forall\delta, c\in\Omega_n^C, \forall n, f=1, o=1, t=1 \quad (66)$$

$$E_{\delta,c,f,o,t}^{TES} = E_{\delta,c,f-1,o=lo(o),t=lt(t)}^{TES} + p_{\delta,c,f,o,t}^{TES,inj} - p_{\delta,c,f,o,t}^{TES,out} \quad \forall\delta, c\in\Omega_n^C, \forall n, f>1, o=1, t=1 \quad (67)$$

$$E_{\delta,c,f,o,t}^{TES} = E_{\delta,c,f,o-1,t=lt(t)}^{TES} + p_{\delta,c,f,o,t}^{TES,inj} - p_{\delta,c,f,o,t}^{TES,out} \quad \forall\delta, c\in\Omega_n^C, \forall n, \forall f, o>1, t=1 \quad (68)$$

$$E_{\delta,c,f,o,t}^{TES} = E_{\delta,c,f,o,t-1}^{TES} + p_{\delta,c,f,o,t}^{TES,inj} - p_{\delta,c,f,o,t}^{TES,out} \quad \forall\delta, c\in\Omega_n^C, \forall n, \forall f, \forall o, t>1 \quad (69)$$

$$0 \leq E_{\delta,c,f,o,t}^{TES} \leq E_{c,f}^{TES,cap} \quad \forall\delta, c\in\Omega_n^C, \forall n, \forall f, \forall o, \forall t \quad (70)$$

$$p_{,c,f,o,t\delta}^{PB} = \iota_c p_{\delta,c,f,o,t}^{TES,out} \quad \forall\delta, c\in\Omega_n^C, \forall n, \forall f, \forall o, \forall t \quad (71)$$

$$0 \leq p_{,c,f,o,t\delta}^{PB} \leq P_{c,f}^{PB,cap} \quad \forall\delta, c\in\Omega_n^C, \forall n, \forall f, \forall o, \forall t \quad (72)$$

HPS Plant Operations

$$p^{H,turbine}_{\delta,h,f,o,t} = \xi_h K^{turbine}_h q^{turbine}_{\delta,h,f,o,t} H_h \qquad \forall\delta, h\epsilon\Omega^H_n, \forall f, \forall o, \forall t \qquad (73)$$

$$p^{H,turbine}_{\delta,h,f,o,t} = \frac{K^{pump}_h q^H_{\delta,h,f,o,t} H_h}{\xi_h} \qquad \forall\delta, h\epsilon\Omega^H_n, \forall n, \forall f, \forall o, \forall t \qquad (74)$$

$$0 \le q^{H,turbine}_{\delta,h,f,o,t} \le V_h V^{H,cap}_{h,f} \qquad \forall\delta, h\epsilon\Omega^H_n, \forall n, \forall f, \forall o, \forall t \qquad (75)$$

$$0 \le q^{H,pump}_{\delta,h,f,o,t} \le V_h V^{H,cap}_{h,f} \qquad \forall\delta, h\epsilon\Omega^H_n, \forall n, \forall f, \forall o, \forall t \qquad (76)$$

$$V^H_{\delta,h,f,o,t} = \mu_h V^{H,inv}_{\delta,h,f,o,t} + q^{H,pump}_{\delta,h,f,o,t} - q^{H,turbine}_{\delta,h,f,o,t} \qquad \forall\delta, h\epsilon\Omega^H_n, \forall n, f=1, o=1, t=1 \qquad (77)$$

$$V^H_{\delta,h,f,o,t} = V^{H,inv}_{\delta,h,f-1,o=lo(o),t=lt(t)} + q^{H,pump}_{\delta,h,f,o,t} - q^{H,turbine}_{\delta,h,f,o,t} \qquad \forall\delta, h\epsilon\Omega^H_n, \forall n, f>1, o=1, t=1 \qquad (78)$$

$$V^H_{\delta,h,f,o,t} = V^{H,inv}_{\delta,h,f,o-1,t=lt(t)} + q^{H,pump}_{\delta,h,f,o,t} - q^{H,turbine}_{\delta,h,f,o,t} \qquad \forall\delta, h\epsilon\Omega^H_n, \forall n, f, o>1, t=1 \qquad (79)$$

$$V^H_{\delta,h,f,o,t} = V^{H,inv}_{\delta,h,f,o,t-1} + q^{H,pump}_{\delta,h,f,o,t} - q^{H,turbine}_{\delta,h,f,o,t} \qquad \forall\delta, h\epsilon\Omega^H_n, \forall n, \forall o, t>1 \qquad (80)$$

$$0 \le V_{\delta,h,f,o,t} \le V^{H,cap}_{h,f} \qquad \forall\delta, h\epsilon\Omega^H_n, \forall n, \forall f, \forall o, \forall t \qquad (81)$$

Battery Plant Operations

$$0 \le p^{B,dchrg}_{\delta,b,f,o,t} \le \epsilon_b E^{B,cap}_{b,f} \qquad \forall\delta, b\epsilon\Omega^B_n, \forall n, \forall f, \forall o, \forall t \qquad (82)$$

$$0 \le p^{B,chrg}_{\delta,b,f,o,t} \le \epsilon_b E^{B,cap}_{b,f} \qquad \forall\delta, b\epsilon\Omega^B_n, \forall n, \forall f, \forall o, \forall \qquad (83)$$

$$E^B_{\delta,b,f,o,t} = \gamma_b E^{B,inv}_{\delta,b,f,o,t} + p^{B,chrg}_{\delta,b,f,o,t,}\zeta^{chrg}_b - \frac{p^{B,dchrg}_{\delta,b,f,o,t}}{\zeta^{dchrg}_b} \qquad \forall\delta, b\epsilon\Omega^B_n, \forall n, \forall f=1, o=1, t=1 \qquad (84)$$

$$E^B_{\delta,b,f,o,t} = \gamma_b E^{B,inv}_{\delta,b,f,o,t} + E^B_{\delta,b,f-1,o=lo(o),t=lt(t)} + p^{B,chrg}_{\delta,b,f,o,t}\zeta^{chrg}_b - \frac{p^{B,dchrg}_{\delta,b,f,o,t}}{\zeta^{dchrg}_b} \qquad \forall\delta, b\epsilon\Omega^B_n, \forall n, f>1, o=1, t=1 \qquad (85)$$

$$E^B_{\delta,b,f,o,t} = E^B_{\delta,b,f,o-1,t=lt(t)} + p^{B,chrg}_{\delta,b,f,o,t}\zeta^{chrg}_b - \frac{p^{B,dchrg}_{\delta,b,f,o,t}}{\zeta^{dchrg}_b} \qquad \forall\delta, b\epsilon\Omega^B_n, \forall n, \forall f, o>1, t=1 \qquad (86)$$

$$E^B_{\delta,b,f,o,t} = E^B_{\delta,b,f,o,t-1} + p^{B,chrg}_{\delta,b,f,o,t}\zeta^{chrg}_b - \frac{p^{B,dchrg}_{\delta,b,f,o,t}}{\zeta^{dchrg}_b} \qquad \forall\delta, b\epsilon\Omega^B_n, \forall n, \forall f, \forall o, t>1 \qquad (87)$$

$$\gamma_b E^{B,cap}_{b,f} \le E^B_{\delta,b,f,o,t} \le E^{B,cap}_{b,f} \qquad \forall\delta, b\epsilon\Omega^B_n, \forall n, \forall f, \forall o, \forall t \qquad (88)$$

Transmission Line Flow

$$F^L_{\delta,l,f,o,t} = a_{l}, fB_l\left(\theta_{\delta,s(l),f,o,t} - \theta_{\delta,r(l),f,o,t}\right) \qquad \forall\delta, \forall l \in \Omega^{L^+}, \forall f, \forall o, \forall t \qquad (89)$$

$$-F^{L,cap}_l \le F^L_{\delta,l,f,o,t} \le F^{L,cap}_l \qquad \forall\delta, \forall l \in \Omega^{L^+}, \forall f, \forall o, \forall t \qquad (90)$$

$$-\pi \le \theta_{\delta,n,f,o,t} \le \pi \qquad \forall\delta, \forall n, \forall f, \forall o, \forall t \qquad (91)$$

$$\theta_{\delta,n,f,o,t} = 0 \qquad n: reference \qquad (92)$$

Renewable Spillage Tracking

$$p^{PV,spill}_{\delta,p,f,o,t} = F^{PV}_{\delta,p,f,o,t} P^{PV,cap}_{p,f} - p^{PV,con}_{\delta,p,f,o,t} \qquad \forall \delta, p \in \Omega^{P}_{n}, \forall n, \forall f, \forall o, \forall t \qquad (93)$$

$$p^{PV,spill}_{\delta,w,f,o,t} = F^{W}_{\delta,w,f,o,t} P^{W,cap}_{p,f} - p^{W,con}_{\delta,w,f,o,t} \qquad \forall \delta, p \in \Omega^{W}_{n}, \forall n, \forall f, \forall o, \forall t \qquad (94)$$

Desalination Operations

$$\psi_z Q^{Z,cap}_{z,f} \le q^{Z}_{\delta,z,o,t} \le Q^{Z,cap}_{z,f} \qquad \forall \delta, z \in \Omega^{Z}_{n}, \forall n, \forall f, \forall o, \forall t \qquad (95)$$

Water Storage Tank Operations

$$0 \le q^{K,in}_{\delta,k,f,o,t} \le \sigma_k S^{K,cap}_{k,f} \qquad \forall \delta, k \in \Omega^{K}_{n}, \forall n, \forall f, \forall o, \forall t \qquad (96)$$

$$0 \le q^{K,out}_{\delta,k,f,o,t} \le \sigma_k S^{K,cap}_{k,f} \qquad \forall \delta, k \in \Omega^{K}_{n}, \forall n, \forall f, \forall o, \forall t \qquad (97)$$

$$S^{K}_{\delta,k,f,o,t} = q^{K,in}_{\delta,k,f,o,t} - q^{K,out}_{\delta,k,f,o,t} \qquad \forall \delta, k \in \Omega^{K}_{n}, \forall n, f = 1, o = 1, t = 1 \qquad (98)$$

$$S^{K}_{\delta,k,f,o,t} = S^{K}_{\delta,k,f-1,o=lo(o),t=lt(t)} + q^{K,in}_{\delta,k,f,o,t} - q^{K,out}_{\delta,k,f,o,t} \qquad \forall \delta, k \in \Omega^{K}_{n}, \forall n, f > 1, o = 1, t = 1 \qquad (99)$$

$$S^{K}_{\delta,k,f,o,t} = S^{K}_{\delta,k,f,o-1,t=lt(t)} + q^{K,in}_{\delta,k,f,o,t} - q^{K,out}_{\delta,k,f,o,t} \qquad \forall \delta, k \in \Omega^{K}_{n}, \forall n, \forall f, o > 1, t = 1 \qquad (100)$$

$$S^{K}_{\delta,k,f,o,t} = S^{K}_{\delta,k,f,o,t-1} + q^{K,in}_{\delta,k,f,o,t} - q^{K,out}_{\delta,k,f,o,t} \qquad \forall \delta, k \in \Omega^{K}_{n}, \forall n, \forall f, \forall o, t > 1 \qquad (101)$$

$$0 \le S^{K}_{\delta,k,f,o,t} \le S^{K,cap}_{k,f} \qquad \forall \delta, k \in \Omega^{K}_{n}, \forall n, \forall f, \forall o, \forall t \qquad (102)$$

Nodal Power Balance

$$\sum\nolimits_{j \in \Omega^{j}_{n}} p^{J}_{\delta,j,f,o,t} + \sum\nolimits_{c \in \Omega^{P}_{n}} p^{PB}_{\delta,c,f,o,t} + \sum\nolimits_{p \in \Omega^{P}_{n}} p^{PV,con}_{\delta,p,f,o,t} + \sum\nolimits_{w \in \Omega^{W}_{n}} p^{W,con}_{\delta,w,f,o,t} + \sum\nolimits_{h \in \Omega^{H}_{n}} p^{H,turbine}_{\delta,w,f,o,t} + \sum\nolimits_{b \in \Omega^{B}_{n}} p^{B,dchrg}_{\delta,w,f,o,t} + \sum\nolimits_{l\ r(l)=n} F^{L}_{\delta,w,f,o,t} = P^{H,pump}_{\delta,h,f,o,t} + \sum\nolimits_{h \in \Omega^{H}_{n}} p^{H,pump}_{\delta,h,f,o,t} + \sum\nolimits_{b \in \Omega^{B}_{n}} p^{B,chrg}_{\delta,b,f,o,t} + \sum\nolimits_{l\ s(l)=n} F^{L}_{\delta,l,f,o,t} + \sum\nolimits_{w \in \Omega^{W}_{n}} q^{Z}_{\delta,z,f,o,t} A^{Z}_{z} \qquad \forall \delta, \forall n, \forall f, \forall o, \forall t \qquad (103)$$

System Water Balance

$$\sum\nolimits_{z \in \Omega^{z}} q^{Z}_{\delta,z,f,o,t} + \sum\nolimits_{k \in \Omega^{K}} q^{K,out}_{\delta,k,f,o,t} = \sum\nolimits_{n} Q^{D}_{\delta,n,f,o,t} + \sum\nolimits_{k \in \Omega^{K}} q^{K,in}_{\delta,k,f,o,t} \qquad \forall \delta, \forall f, \forall o, \forall t \qquad (104)$$

Limit on Renewable Generation

$$\psi^{gen} \left(\sum\nolimits_{o} \alpha_o \sum\nolimits_{t=1}^{24} \left[\sum\nolimits_{j \in \Omega^{J}} p^{J}_{\delta,j,f,o,t} + \sum\nolimits_{\substack{j \\ c \in \Omega^{C}_{n}}} p^{PB}_{\delta,j,f,o,t} + \sum\nolimits_{p \in \Omega^{P}_{n}} F^{PV,cap}_{\delta,j,f,o,t} + \sum\nolimits_{\omega \in \Omega^{W}_{n}} F^{W}_{\delta,\omega,f,o,t} P^{W,cap}_{\omega} + \sum\nolimits_{h \in \Omega^{H}_{n}} p^{H,turbine}_{\delta,h,f,o,t} \right] \right) \le \left(\sum\nolimits_{o} \alpha_o \sum\nolimits_{t=1}^{24} \left[\sum\nolimits_{c \in \Omega^{C}_{n}} p^{PB}_{\delta,c,f,o,t} + \sum\nolimits_{p \in \Omega^{P}_{n}} F^{PV}_{\delta,j,f,o,t} P^{PV,cap}_{s,f} + \sum\nolimits_{\omega \in \Omega^{W}_{n}} F^{W}_{\delta,\omega,f,o,t} P^{W,cap}_{w,f} + \sum\nolimits_{h \in \Omega^{H}_{n}} p^{H,turbine}_{\delta,j,f,o,t} \right] \right) \qquad \forall \delta, \forall f \qquad (105)$$

Linearization of Thermal Generation Constraints

We model Equation 53 with the disjunction in Equation 105, where U is a Boolean variable that is true if $u_{\delta,j,f,o,t} = 1$ $(w_{\delta,j,f,o,t} = P_{j,f}^{J,cap})$ and false if $u_{\delta,j,f,o,t} = 0$ $(w_{\delta,j,f,o,t} = 0)$.

$$\begin{bmatrix} U \\ P_{j,f}^{J,cap} - w_{\delta,j,f,o,t} \leq 0 \\ -w_{\delta,j,f,o,t} + P_{j,f}^{J,cap} \leq 0 \end{bmatrix} \vee \begin{bmatrix} \neg U \\ w_{\delta,j,f,o,t}^{Cap} \leq 0 \\ -w_{\delta,j,f,o,t}^{Cap} \leq 0 \end{bmatrix} \quad \forall\delta, \forall j, \forall f, \forall o, \forall t \quad (106)$$

The disjunction in Equation 105 is reformulated as

$$w_{\delta,j,f,o,t} - P_{j,f}^{J,cap} \leq M_1(1 - u_{\delta,j,f,o,t}) \quad \forall\delta, \forall j, \forall f, \forall o, \forall t \quad (107)$$

$$P_{j,f}^{J,cap} - w_{\delta,j,f,o,t} \leq M_2(1 - u_{\delta,j,f,o,t}) \quad \forall\delta, \forall j, \forall f, \forall o, \forall t \quad (108)$$

$$w_{\delta,j,f,o,t} \leq M_3 u_{\delta,j,f,o,t} \quad \forall\delta, \forall j, \forall f, \forall o, \forall t \quad (109)$$

$$-w_{\delta,j,f,o,t} \leq M_4 u_{\delta,j,f,o,t} \quad \forall\delta, \forall j, \forall f, \forall o, \forall t \quad (110)$$

where M_1, M_2, M_3, M_4 are constant values.

By inspection we can deduce that the value of the $M$are:

$$M_1 = 0 \quad (111)$$

$$M_2 = P_{j,f}^{MAX} \quad (112)$$

$$M_3 = P_{j,f}^{MAX} \quad (113)$$

$$M_4 = 0 \quad (114)$$

Linearization of Power Flow Constraints

We model Equation 89 with the disjunction in Equation 115 where A is a Boolean variable that is true if $a_{l,f} = 1$ and false if $a_{l,f} = 0$.

$$\begin{bmatrix} A \\ -B_l(\theta_{\delta,s(l),f,o,t} - \theta_{\delta,r(l),f,o,t}) + F_{\delta,l,f,o,t}^L \leq 0 \\ B_l(\theta_{\delta,s(l),f,o,t} - \theta_{\delta,r(l),f,o,t}) - F_{\delta,l,f,o,t}^L \leq 0 \\ F_{\delta,l,f,o,t}^L - F_l^{L,max} \leq 0 \\ -F_{\delta,l,f,o,t}^L + F_l^{L,max} \leq 0 \end{bmatrix} \vee \begin{bmatrix} \neg A \\ F_{\delta,l,f,o,t}^L \leq 0 \\ -F_{\delta,l,f,o,t}^L \leq 0 \\ -F_{\delta,l,f,o,t}^L \leq F_l^{L,max} \\ F_{\delta,l,f,o,t}^L \leq 0 \end{bmatrix} \quad (115)$$

$$\forall\delta, \forall l, \forall f, \forall o, \forall t$$

The disjunction in Equation 115 is reformulated as

$$F_{\delta,l,f,o,t}^L - B_l(\theta_{\delta,s(l),f,o,t} - \theta_{\delta,r(l),f,o,t}) \leq M_1(1 - a_{l,f}) \quad \forall\delta, \forall l, \forall f, \forall o, \forall t \quad (116)$$

$$-F_{\delta,l,f,o,t}^L + B_l(\theta_{\delta,s(l),f,o,t} - \theta_{\delta,r(l),f,o,t}) \leq M_2(1 - a_{l,f}) \quad \forall\delta, \forall l, \forall f, \forall o, \forall t \quad (117)$$

$$F^{L}_{\delta,l,f,o,t} - F^{L,max}_{l} \leq M_3(1 - a_{l,f}) \quad \forall\delta, \forall l, \forall f, \forall o, \forall t \tag{118}$$

$$-F^{L}_{\delta,l,f,o,t} - F^{L,max}_{l} \leq M_4(1 - a_{l,f}) \quad \forall\delta, \forall l, \forall f, \forall o, \forall t \tag{119}$$

$$F^{L}_{\delta,l,f,o,t} \leq M_5 a_{l,f} \quad \forall\delta, \forall l, \forall f, \forall o, \forall t \tag{120}$$

$$-F^{L}_{\delta,l,f,o,t} \leq M_6 a_{l,f} \quad \forall\delta, \forall l, \forall f, \forall o, \forall t \tag{121}$$

where $M_1, M_2, M_3, M_4, M_5, M_6$ are constant values.

By inspection we can deduce that the value of the M are:

$$M_1 = \pi \tag{122}$$

$$M_2 = \pi \tag{123}$$

$$M_3 = 0 \tag{124}$$

$$M_4 = -F^{L,max}_{l} \tag{125}$$

$$M_5 = F^{L,max}_{l} \tag{126}$$

$$M_6 = -F^{L,max}_{l} \tag{127}$$

We simplify the previous equations as

$$F^{L}_{\delta,l,f,o,t} - B_l\big(\theta_{\delta,s(l),f,o,t} - \theta_{\delta,r(l),f,o,t}\big) \leq \pi(1 - a_{l,f}) \quad \forall\delta, \forall l, \forall f, \forall o, \forall t \tag{128}$$

$$-F^{L}_{\delta,l,f,o,t} + B_l\big(\theta_{\delta,s(l),f,o,t} - \theta_{\delta,r(l),f,o,t}\big) \leq \pi(1 - a_{l,f}) \quad \forall\delta, \forall l, \forall f, \forall o, \forall t \tag{129}$$

$$F^{L}_{\delta,l,f,o,t} \leq F^{L,max}_{l} a_{l,f} \quad \forall\delta, \forall l, \forall f, \forall o, \forall t \tag{130}$$

$$-F^{L}_{\delta,l,f,o,t} \leq -F^{L,max}_{l} a_{l,f} \quad \forall\delta, \forall l, \forall f, \forall o, \forall t \tag{131}$$

Supplementary note 7: Levelized Cost Calculations
Levelized cost of electricity (LCOE)
Levelized cost of electricity[9] measures the average net present cost of electricity generation over the useful lifetime of a generating technology. In this case, the lifetime is the duration of the planning horizon. LCOE is a ratio between the discounted sum of total investment costs (TIC) and the total operating cost (TOC) divided by the discounted sum of the total power produced (TPP).

$$LCOE = \frac{\sum_f \frac{TIC_f^{power} + TOC_f^{power}}{(1+r)^f}}{\sum_f \frac{TPP_f^{power}}{(1+r)^f}} \tag{132}$$

$$TIC_f^{power} = \sum_{b\in\Omega^B} I_{b,f}^B P_{b,f}^{B,inv} + \sum_{c\in\Omega^C} I_{c,f}^{PB} P_{c,f}^{PB,inv} + \sum_{h\in\Omega^H} I_{hf}^H \left(K_h^{turbine} v_h V_{h,f}^{H,inv} H_h\right) + \sum_{j\in\Omega^J} I_{j,f}^J P_{j,f}^{J,inv} + \sum_{p\in\Omega^P} I_{p,f}^{PV} P_{p,f}^{PV,inv} + \sum_{w\in\Omega^W} I_{w,f}^W P_{w,f}^{W,inv} \tag{133}$$

$$TOC_f^{power} = \sum_\delta \beta_\delta \sum_o \alpha_o \sum_{t=1}^{24} \Big[\sum_{b\in\Omega^B} C_b^B \left(E_{\delta,b,f,o,t}^{B,charge} + E_{\delta,b,f,o,t}^{B,discharge}\right) + \sum_{c\in\Omega^C} C_c^{PB} p_{\delta,c,f,o,t}^{PB} + \sum_{h\in\Omega^H} C_h^H \left(p_{\delta,h,f,o,t}^{H,pump} + p_{\delta,h,f,o,t}^{turbine}\right) + \sum_{j\in\Omega^J} C_{\delta,j,f,o,t}^J p_{j,f}^{J,inv} + \sum_{p\in\Omega^P} C_p^{PV} F_{\delta,p,f,o,t}^{PV} P_{\delta,p,f,o,t}^{PV,cap} + \sum_{w\in\Omega^W} C_w^W F_{\delta,w,f,o,t}^W P_{\delta,w,f,o,t}^{W,cap}\Big] \tag{134}$$

$$TPP_f^{power} = \sum_\delta \beta_\delta \sum_o \alpha_o \sum_{t=1}^{24} \Big[\sum_{c\in\Omega^C} p_{\delta,c,f,o,t}^{PB} + \sum_{h\in\Omega^H} \left(p_{\delta,h,f,o,t}^{H,pump} + p_{\delta,h,f,o,t}^{turbine}\right) + \sum_{j\in\Omega^J} p_{j,f}^{J,inv} + \sum_{p\in\Omega^P} \left(F_{\delta,p,f,o,t}^{PV} P_{\delta,p,f,o,t}^{PV,cap} - p_{\delta,p,f,o,t}^{PV,spill}\right) + \sum_{w\in\Omega^W} \left(F_{\delta,w,f,o,t}^W P_{\delta,w,f,o,t}^{W,cap} - P_{\delta,w,f,o,t}^{W,spill}\right)\Big] \tag{135}$$

Levelized cost of water (LCOW)
Levelized cost of water[3] measures the average net present cost of water production over the useful lifetime of a generating technology. In this case, the lifetime is the duration of the planning horizon. LCOW is a ratio between the discounted sum of total investment costs (TIC) and the total operating cost (TOC) divided by the discounted sum of the total power produced (TPP).

$$LCOW = \frac{\sum_f \frac{TIC_f^{water} + TOC_f^{water}}{(1+r)^f}}{\sum_f \frac{TPP_f^{water}}{(1+r)^f}} \tag{136}$$

$$TIC_f^{water} = \sum_{k\in\Omega^K} I_{k,f}^K S_{k,f}^{K,inv} + \sum_{z\in\Omega^Z} I_{z,f}^Z Q_{z,f}^{Z,inv} \tag{137}$$

$$TOC_f^{water} = \sum_\delta \beta_\delta \sum_o \alpha_o \sum_{t=1}^{24} \Big[\sum_{k\in\Omega^k} C_k^k \left(q_{\delta,k,f,o,t}^{K,in} + q_{\delta,k,f,o,t}^{K,out}\right) + \sum_{z\in\Omega^z} C_z^Z p_{\delta,k,f,o,t}^{PB}\Big] \tag{138}$$

$$TPP_f^{water} = \sum_\delta \beta_\delta \sum_o \alpha_o \sum_{t=1}^{24} \Big[\sum_{z\in\Omega^z} p_{\delta,k,f,o,t}^{PB}\Big] \tag{139}$$

Supplementary note 8: The expected value of perfect information and the value of the stochastic solution[10]

The Expected Value of Perfect Information (EVPI)

A general two-stage stochastic programming problem can be represented as:

$$\min_{x,y} \mathrm{E}[\mathbf{c}^T\mathbf{x} + \boldsymbol{q}(\xi)^T\boldsymbol{y}(\xi)] \quad (140)$$

s.t

$$\mathbf{Ax} = \mathbf{b} \quad (141)$$

$$\mathbf{T}(\xi)\mathbf{x} + \mathbf{W}(\xi)\mathbf{y}(\xi) = \mathbf{h}(\xi) \quad (142)$$

$$\mathbf{x} \in X, \mathbf{y}(\xi) \in Y, \quad (143)$$

where $\boldsymbol{\xi}$ is a random variable with a certain probability distribution. We address the solution of problem (139)-(142) assuming discrete realizations of the random variable ξ, the so-called discrete scenarios. We denote the optimal objective function of problem (139)-(142) as SP.

To calculate the EVPI, we introduce the wait-and-see solution (WS). The WS solution is obtained by solving problem (139)-(142) for each discrete scenario individually. We denote the optimal solution of each problem as $x_s^*, \forall s \in S,$ where s is a scenario index and S is the set of scenarios, and W_s as the corresponding optimal objective function value.
The wait-and-see solution is defined as

$$WS = \mathrm{E}[W_s]. \quad (144)$$

Based on this notation, the EVPI is defined as:

$$EVPI = SP - WS. \quad (145)$$

The EVPI represents the maximum payment one would be willing to make to obtain perfect information.

The Value of the Stochastic Solution (VSS)

To calculate the VSS, we first calculate the expected value problem, defined by using the average of the random variables in problem (19)-(142) instead of the multiple scenarios. The optimal solution of this problem is denoted by the expected value solution x^{EV}. Then we solve problem (139)-(142) with x fixed to x^{EV}considereing the discrete scenarios. The optimal objective function value is denoted by EEV. Based on this notation, the VSS is defined as

$$VSS = EEV - SP. \quad (146)$$

The VSS represents the cost of ignoring uncertainty when planning.

However, for some problems, such as the one presented in this manuscript, using the EEV solution, x^{EV}, may result in infeasibilities for some scenarios. In this case, the value of EEV is positive infinity. However, this answer does not say much about the VSS.[10] For these cases, using the worst-case solution, x^{wc} , obtained from solving problem (139)-(142) for the worst-case scenario only (given that it can be identified) is an alternative. Then we solve problem (139)-(142) with x fixed to x^{wc} considering the discrete scenarios. The optimal objective

function value is denoted as the expected value of the reference scenario (EVRS) and the VSS is defined as

$$VSS = EVRS - SP. \tag{147}$$

The results for the SP, WS, EVRS, EVPI and VSS, are presented in Supplementary Table 7.

Supplementary note 9: Comparison of Representative days vs. Full-time Resolution

This note provides a comparison between time representations in the planning model used in this study: 1) representative days, each with an hourly resolution, used to approximate weather and demand conditions over one year; and 2) using a full-time resolution with 8760 hours per year. The use of representative days in the stochastic programming models is motivated by the expected high computational cost of models using a full-time resolution over a multi-year horizon. To compare the performance of the proposed model using representative days versus a full-time resolution model, we designed three computational experiments:

1. Comparing stochastic programming models using a) representative days; and b) a full-time resolution; considering a time horizon with ten years.
2. Comparing stochastic programming model using a) representative days; and b) a full-time resolution; considering a time horizon with one year.
3. Comparing deterministic models using a) representative days; and b) a full-time resolution; considering a time horizon with one year.

In the first experiment, we contrast the results discussed in the main manuscript text with the ones from a stochastic programming model for ten years using the full hourly resolution (8760 hours) for the base case. We specified a computational time limit of 14 days, and when the time limit was reached, we obtained a final optimality gap of 43.2%. Thus, in this case we do not have a certificate of global optimality, and consequently cannot consider the results to be close to the optimal solution. The inability to solve the full-time resolution ten-year model after 14 days (336 hours) of computation time shows the limitations of a full-time resolution model in a stochastic setup and a multi-year horizon The representative period model presented in the results section of the manuscript solved the model in under seven hours and was able to close the optimality gap, which highlights the advantages of adopting representative days. Supplementary Table 18 summarizes the computation time and optimality gap for the two models.

In the second experiment, for a clearer comparison between a stochastic programming model with a representative period approach and full-time hourly resolution approach, we run for only the final year, 2029. By doing so, we reduce the model size while still capturing the amount of capacity necessary by the end of the time horizon; a common practice found in the literature.[11–15] The downside of running the model for only the last year is the inability to determine when

along the time horizon a technology would be installed; rather we obtain a snapshot of the what the system will look like.

Supplementary Table 19 shows that using the stochastic programming model with a full-time resolution, there is an increase in CSP and wind capacity; 5.12% and 2.21% more of the respective capacities compared to the representative day model. In this case, the additional capacity is attributed to infrequent hours throughout the year that have high power demand and low available renewable resources. To meet power demand a slightly higher capacity is needed. Because the representative day model uses periods that best represent an entire year, days that contain these outliers were not selected. Nevertheless, the representative day model gives a very close approximation to the generation mix to that obtained using a full-time resolution model. The close agreement between the two models, together with the decreased computation time that a representative day model offers, is indicative of the benefits of using the latter approach.

In the third experiment, we focus on one-year deterministic runs using the mean power and water demand data. Supplementary Table 20 presents similar results to those generated in the analysis of the stochastic models. The full-time resolution model suggests additional CSP and wind capacity; the model indicates 3% and 1.64% more of the respective technologies. This further highlight that adopting a representative day approach is quite advantageous in obtaining a robust approximation of the optimal decision.

It is important to note that for the deterministic model, we assume perfect information meaning that we assume the power and water demands are certain. However, with planning models we know that there is no perfect information concerning demands occurring many years in the future. In the case that the demand realizations are much higher than the mean scenario, the built system would be unable to produce sufficient water and electricity. In contrast, a stochastic optimization approach would allow the decision-maker to build a system that would be feasible for every scenario envisioned.

Supplementary References

1. U.S. Energy Information Adminstration. Homepage. Available at: https://www.eia.gov/. (Accessed: 10th March 2021)
2. National Renewable Energy Laboratory. Homepage. (2014). Available at: https://www.nrel.gov/. (Accessed: 10th March 2021)
3. Caldera, U., Bogdanov, D., Afanasyeva, S. & Breyer, C. Role of seawater desalination in the management of an integrated water and 100% renewable energy based power sector in Saudi Arabia. *Water (Switzerland)* **10**, (2017).
4. Lloyd, S. P. Least Squares Quantization in PCM. *IEEE Trans. Inf. Theory* **28**, 129–137 (1982).
5. Ostrovsky, R., Rabani, Y., Schulman, L. J. & Swamy, C. The effectiveness of Lloyd-type methods for the k-means problem. *J. ACM* **59**, 1–22 (2012).
6. Arthur, D. & Vassilvitskii, S. K-means++: The advantages of careful seeding. *Proc. Annu. ACM-SIAM Symp. Discret. Algorithms* **07-09-Janu**, 1027–1035 (2007).
7. Vestas. Homepage. Available at: https://www.vestas.com/. (Accessed: 11th March 2021)
8. Lorenzo, E. *Energy Collected and Delivered by PV Modules*. *Handbook of Photovoltaic Science and Engineering* (2011). doi:10.1002/9780470974704.ch22
9. Short, W., Packey, D. & Holt, T. A manual for the economic evaluation of energy efficiency and renewable energy technologies. *Renew. Energy* **95**, 73–81 (1995).
10. Birge, J. & Louveaux, F. *Stochastic Programming.* **49**, (1998).
11. Alraddadi, M., Conejo, A. J. & Lima, R. M. Expansion Planning for Renewable Integration in Power System of Regions with Very High Solar Irradiation. *J. Mod. Power Syst. Clean Energy* **9**, 485–494 (2021).
12. Baringo, L. & Conejo, A. J. Correlated wind-power production and electric load scenarios for investment decisions. *Appl. Energy* **101**, 475–482 (2013).
13. Domínguez, R., Conejo, A. J. & Carrión, M. Toward fully renewable electric energy systems. *IEEE Trans. Power Syst.* **30**, 316–326 (2015).
14. Almansoori, A. & Shah, N. Design and operation of a stochastic hydrogen supply chain network under demand uncertainty. *Int. J. Hydrogen Energy* **37**, 3965–3977 (2012).
15. Xu, Q., Li, S. & Hobbs, B. F. Generation and storage expansion co-optimization with consideration of unit commitment. *2018 Int. Conf. Probabilistic Methods Appl. to Power Syst. PMAPS 2018 - Proc.* 1–6 (2018). doi:10.1109/PMAPS.2018.8440205
