# Supplementary material for: Simulated co-optimization of renewable energy and desalination systems in Neom, Saudi Arabia
Source: Nat Commun. 2022 Jun 18;13:3514. doi: 10.1038/s41467-022-31233-3 (PMC9206678; doi:10.1038/s41467-022-31233-3)
Supplement: Supplementary file 2 — Description of Additional Supplementary Information [file 41467_2022_31233_MOESM2_ESM.pdf]

Description of Additional Supplementary Information

Title: Supplementary Data 1

Description: Supporting data file for Figures 2, 3, 4, and 5.

Title: Supplementary Data 2

Description: Supporting data file with representative days and regions, and capacity factors use as input data in the optimization model.
